# N-Hydroxyalkyl and 4-Substituted-N-(hydroxyhexyl)-1,8-naphthalimides: Synthesis and Impact of Molecular Structure on Electrochemical and Photophysical Properties

**DOI:** 10.3390/molecules31071178

**Published:** 2026-04-02

**Authors:** Ahmed Chelihi, Ammara Aslam, Krzysztof Karoń, Wojciech Szczepankiewicz, Anna Korytkowska-Wałach, Krzysztof Walczak, Przemyslaw Ledwon

**Affiliations:** 1 Laboratory of Catalysis and Synthesis in Organic Chemistry, University of Tlemcen, Tlemcen 13000, Algeria; 2 Department of Organic Chemistry, Bioorganic Chemistry and Biotechnology, Silesian University of Technology, Krzywoustego 4, 44-100 Gliwice, Poland; 3 Department of Physical Chemistry and Technology of Polymers, Faculty of Chemistry, Silesian University of Technology, 9. M. Strzody St., 44-100 Gliwice, Poland; 4 PhD School, Silesian University of Technology, 2a Akademicka Str., 44-100 Gliwice, Poland

**Keywords:** 1,8-naphthalimides, structure–property relationship, electrochemical properties, branched hydroxyalkyl, electron-donating substituents, electron-withdrawing substituents

## Abstract

Two series of *N*-hydroxyalkyl-1,8-naphthalimide derivatives were synthesized to investigate the influence of structural variables on their electrochemical and photophysical properties. The first series includes compounds containing N-hydroxyalkyl substituents of various chain lengths and branches. The second series includes derivatives functionalized in the naphthalene core with electron-donating or electron-withdrawing groups. Cyclic voltammetry, UV–Vis spectroscopy and fluorescence measurements were supported by theoretical DFT calculations. Branched hydroxyalkyl chains enhanced photoluminescence quantum yields by up to 19%, compared to less than 4% for linear chains. Functionalization of the naphthalene core at the C4 position strongly affected optical band gaps, electrochemical properties and photoluminescence quantum yields. DFT calculations revealed significant changes in the energies of the frontier orbits: the HOMO energy varied from −6.95 eV to −5.51 eV, while the LUMO energy varied from −3.24 eV to −1.94 eV. Preliminary tests have demonstrated the suitability of the selected derivatives as cathode materials in lithium-ion batteries, achieving an initial capacity of 47 mAh/g.

## 1. Introduction

1,8-Naphthalimide (NI) derivatives have attracted the attention of scientists for the last fifty years because of their tunable photophysical and electrochemical properties [1,2,3]. The presence of an aromatic π-electron system conjugated with electron-withdrawing carbonyl groups of the imide type results in a π-deficient character of the aromatic skeleton. Although synthetic routes to NI derivatives are well understood, several aspects still require further investigation and refinement. Common groups like nitro, sulpho or halogen atoms can be introduced easily under mild conditions through electrophilic aromatic substitution. Introduction of other substituents is possible through nucleophilic substitution of these groups or direct C-C bond formation [1,2,4].

Systematic studies correlating specific structural features, such as hydroxyalkyl chain topology or the electronic effects of substituents, with their optical and electrochemical properties remain lacking in the literature. In particular, the role of intramolecular hydrogen bonds, steric hindrance, and mesomeric/inductive effects in controlling fluorescence and HOMO–LUMO energy gaps has not been fully elucidated. Such modifications influence electrochemical and photophysical properties, including absorption and emission wavelengths, fluorescence quantum yields (QY), and redox potentials, which make these compounds attractive for various applications such as optoelectronic devices, sensors, energy storage, emissive materials [1,5,6,7,8,9,10,11,12], cell imaging [13,14,15,16] and anticancer agents [17]. Moreover, NI derivatives have been utilized as a class of dyes with strong fluorescence and excellent photo stability, which makes them particularly valuable as fluorescent probes for different targets [18,19,20,21].

One of the most frequently modified properties of organic dyes is the wavelength of their emitted light [22,23,24]. For example, various fluorescent probes based on NI have been developed and then used for the detection of various analytes [14]. In this case, the main factors that favor the use of these compounds are their very good photostability and fluorescence efficiency. However, fluorescence QY varies significantly between different NI derivatives because of interactions with various substituents. When used as emissive materials in organic light-emitting diodes, NI derivatives exhibit different emission wavelengths [18]. Substitutions at the C4 position allow for extensive modifications of the emitted wavelength and lead to the possibility of obtaining derivatives that emit light in a wide range, including blue, green, and red [25]. Also, the flexibility of the side groups has been reported as a factor influencing emission properties, and especially QY [26].

The presence of electron-donating groups on the electron-deficient 1,8-naphthalimide core leads to a donor–π–acceptor (D–π–A) dipolar architecture, increasing the conjugation system and greatly enhancing the fluorescence intensity of this system structure, which is thus being widely used in the design of new fluorescent dyes [15,27,28,29,30]. Such “push–pull” systems involve the formation of an intramolecular charge-transfer (ICT) excited state and result in significant bathochromic shifts in both absorption and emission spectra.

The electrochemical properties of 1,8-naphthalimides are often studied due to the possibility of correlating the oxidation and reduction potentials of these compounds with their HOMO and LUMO orbital energies [31]. For this purpose, cyclic voltammetry, in particular, is often used, which is the basic method for studying electroactive compounds. 1,8-Naphthalimide derivatives are often easily reduced due to the presence of carbonyl bonds within them. In addition, the reduction process in some of the derivatives has been shown to be reversible. This is an important feature due to the current extensive research on electroactive materials for applications in rechargeable batteries and redox flow batteries [32,33,34]. In this respect, there are only a few reports in the literature about research conducted on this group of compounds.

Herein, we focus on synthesis and structure–property relationships in two series of 1,8-naphthalimides in which N-hydroxyalkyl substitutions of various lengths and branches, and functionalization by different electron-donating and electron-withdrawing groups, are investigated. Optical and electrochemical properties were determined by UV–Vis, fluorescence spectroscopy and cyclic voltammetry measurements. The experimental data are compared with Density Functional Theory (DFT) calculations.

## 2. Results and Discussion

### 2.1. Chemistry

#### 2.1.1. Synthesis of N-Hydroxyalkyl-1,8-naphthalimide Derivatives

**Scheme 1:**
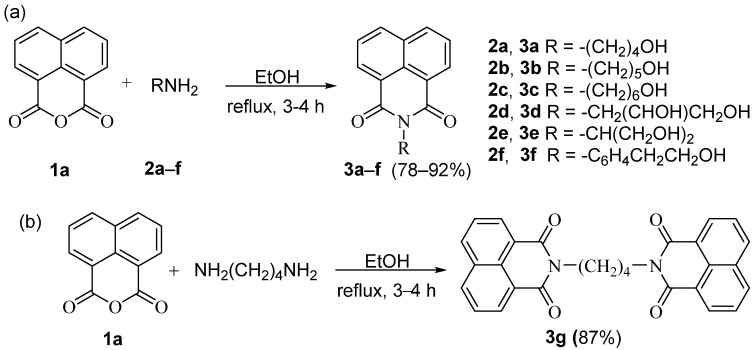
(**a**) Synthesis of N-hydroxyalkyl or N-(dihydroxy)alkyl derivatives of 1,8-naphthalimide **3a**–**f**. (**b**) Condensation of 1,8-naphthalic anhydride with 1,4-diaminobutane.

Commercially available 1,8-naphthalic anhydride **1a** was subjected to reaction with appropriate ω-amino alcohols **2a**–**2c**, **2f** possessing a hydroxyl group on the terminal carbon atom of the alkyl chain or aminodiols **2d**–**2e**. The anhydride and an equimolar amount of amino alcohol were refluxed in ethanol (EtOH). The progress of the reaction was monitored by thin-layer chromatography (TLC), using CHCl_3_/CH_3_OH (95:5, *v*/*v*) as the eluent. After decay of the substrate, the reaction mixture was cooled to room temperature, and the precipitated solid was filtered off, washed with distilled water and recrystallized from absolute ethanol. Products **3a**–**3f** were isolated in excellent yields (Scheme 1a). In another experiment, anhydride **1a** (2 eq.) with 1,4-butanediamine **2g** in boiled ethanol gave a symmetrical 2,2′-(butane-1,4-diyl)bis(1*H*-benzo[*de*]isoquinoline-1,3(2*H*)-dione) **3g** in 87% yield (Scheme 1b).

**Scheme 2:**
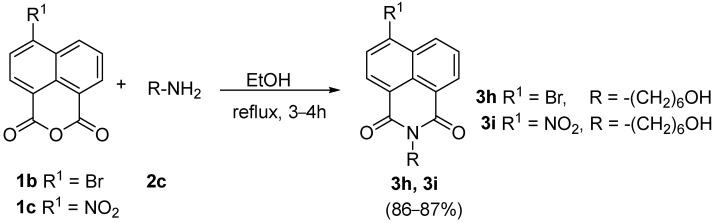
Synthesis of 1,8-naphthalimide derivatives **3h**–**i**.

It is well known that the introduction of labile groups like bromo or nitro groups on carbon C4 in the 1,8-naphthalic anhydride nucleus is possible when starting from acenaphthene, whereas bromination or nitration of 1,8-naphthalic anhydride leads to the introduction of a substituent in 3 positions due to the directing effect of carbonyl groups present in the anhydride molecule [35]. Thus, **1b** was prepared following the procedures elaborated in [36], where acenaphthene was brominated using N-bromosuccinimide (NBS) in N,N-dimethylformamide (DMF) at room temperature, followed by oxidation with potassium dichromate. Similarly, nitration of 1,8-naphthalic anhydride with nitric acid in glacial acetic acid at room temperature, followed by oxidation with potassium dichromate, led to the formation of 4-nitro-1,8-naphthalic anhydride **1c**. The desired compounds, 4-substituted 1,8-naphthalic anhydride **1b** and **1c**, were obtained in 63% and 46% yields, respectively (not depicted in Scheme 2) [36]. The 4-bromo- or 4-nitro-1,8-naphthalic anhydrides **1b** and **1c** were converted into N-(6-hydroxyhexyl)-1,8-naphthalimides **3h**–**3i** by treatment with 6-aminohexanol **2c** in ethanol under reflux. Products **3h**–**3i** were isolated in good yields of 85–87% after 3-4 h of reaction time. (Scheme 2). The results of the preparation of 4-bromo- or 4-nitro-1,8-naphthalic anhydrides and N-hydroxyalkyl-1,8-naphthalimide derivatives are summarized in Table S1.

#### 2.1.2. Synthesis of New 4-Substituted N-Hydroxyalkyl-1,8-naphthalimide Derivatives

To the best of our knowledge, among the planned 4-substituted N-(hydroxyalkyl)-1,8-naphthalimides, only compound **5b** has previously been reported [37,38]. According to Zhang [37] the substitution of a bromine atom by piperidine was carried out in 2-methoxyethanol at 110 °C using 2-(6-hydroxyhexyl)-1*H*-benzo[*de*]isoquinoline-1,3(2*H*)-dione as reactant; the yield of the product is not reported. In a second work, a different approach was applied. Treatment of 4-bromo-1,8-naphthalic anhydride with piperidine in a 2-methoxyethanol solution at 125 °C for 4–5 h gave 4-piperidin-1-yl-1,8-naphthalic anhydride with a yield of 75% [38]. Condensation with 6-amino-1-hexanol gave the desired imide in a yield of up to 70%. The physicochemical data were not reported. In our strategy, we seek universal conditions that are applicable to all planned derivatives.

Our synthetic strategy for preparing 4-substituted N-hydroxyalkyl-1,8-naphthalimide derivatives focused on evaluating various reaction parameters, including solvent type, reaction time, and product isolation methods. Synthesis of **5a** from **3h** was selected as a model reaction to develop a general experimental procedure for synthesizing a wider group of derivatives. This systematic approach enabled us to identify an optimal reaction pathway that provides high yields within very short reaction times under conditions consistent with the principles of green chemistry.

Table 1 summarizes the results. We observed that the reaction proceeds efficiently only in the presence of a tertiary, non-nucleophilic base. Organic bases with even weak nucleophilicity led to competing side reactions, as they can act as nucleophiles and participate in substitution processes. This undesired involvement reduces selectivity toward substitution at the 4-position of the 1,8-naphthalimide core. In contrast, non-nucleophilic tertiary bases exclusively deprotonate the N-hydroxyalkylamine, generating active nucleophilic species without interfering with the reaction pathway. This selectivity is essential for promoting nucleophilic aromatic substitution at the activated 4-position, which is rendered electrophilic by the strong electron-withdrawing imide groups. Consequently, utilizing triethylamine (Et_3_N) (entries 9) as a non-nucleophilic tertiary base ensures clean substitution, minimizes side reactions, and leads to higher yields of the target derivatives of 4-substituted N-hydroxyalkyl-1,8-naphthalimide (**3h**–**3i**).

The use of 4-pyrrolidinylpyridine (4-PPy) as a base in DMF or 1,4-dioxane resulted in low yields (50–60%), as 4-PPy is highly reactive, expensive, and less selective (entries 4–5). Furthermore, in polar protic solvents, 4-PPy significantly reduced selectivity, leading to a mixture of products; among them, the desired product was missing (entry 3). In contrast, 0.03 mL of 1,8-diazabicyclo [5.4.0]undec-7-ene (DBU) afforded high yields (80–88%) after 3 h in polar aprotic solvents such as 1,4-dioxane and DMF (entries 6–7). Using potassium carbonate as an inorganic base gave a moderate yield of 70% after 4 h (entry 8). Finally, triethylamine was identified as the optimal base (entry 9), providing an 88% yield after only 2 h. This method is more selective, cost-effective, and requires a considerably shorter reaction time compared to other bases. Among the solvents tested, DMF gave the best results, likely due to its favorable solubility for both the substrate and the catalyst.

**Scheme 3:**
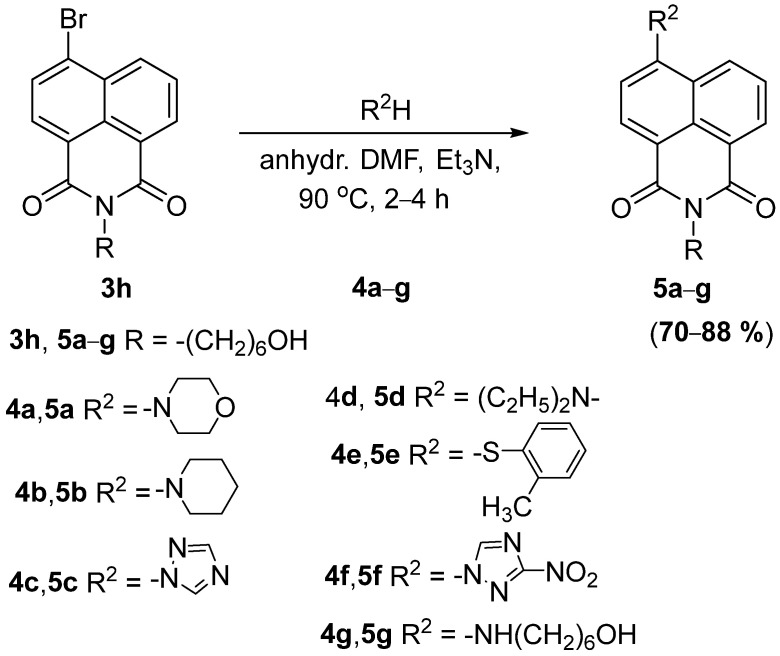
Synthesis of 4-substituted-N-(ω-hydroxyhexyl)-1,8-naphthalimides **5a**–**g**.

The selected conditions in entry 9 were efficiently applied to carry out the subsequent reactions by treating compound **3h** with different N- or S-centered nucleophiles, **4a**–**4g**, in a solution of anhydrous DMF containing triethylamine (Scheme 3). Reaction was conducted at 90 °C and monitored by TLC (CHCl_3_:CH_3_OH, 20/80 *v*/*v*). Once the reaction was completed, the mixture was poured into 50 mL of water. A series of three liquid–liquid extractions was then performed, using water and chloroform for each extraction to remove DMF, thus allowing the target compound, which is soluble in chloroform, to separate. Products **5a**–**5g** were obtained in good yield. The structure of the obtained derivatives was confirmed using NMR and MS spectrometry (please see the Supplementary Information).

### 2.2. DFT Calculations

DFT calculations were employed to determine the molecular geometry of the studied 1,8-naphthalimide derivatives. The computations were carried out using the B3LYP/6-31G(d,p) model. This model was selected because it has been widely employed for similar classes of compounds [39,40,41]. For the calculations, compound **3c** was selected as the unsubstituted reference at the C4 position, together with a group of compounds bearing various electron-donating and electron-withdrawing substituents at this position.

**Figure 1:**
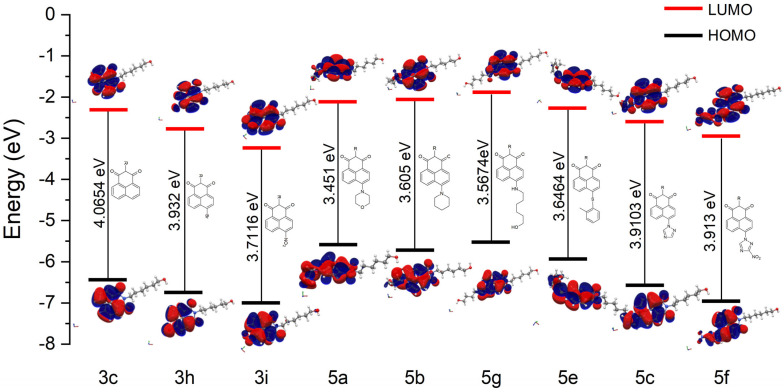
Diagram representing the DFT-calculated energy levels of the selected compounds and the graphical representations of their HOMO and LUMO contours. Calculations were performed using the ORCA 6.0.1 program with the B3LYP functional employing the 6-31G(d,p) basis set, and the 6-311G(d,p) basis set for **3h**.

Based on the optimized geometries, molecular orbital analyses were performed to evaluate the energy levels of the frontier orbitals. A diagram representing the DFT-calculated energy levels of the selected compounds, along with graphical representations of the HOMO and LUMO contours, is presented in Figure 1. The key DFT-calculated parameters are summarized in Table S3, while the full graphical representations of the calculated orbitals are provided in Tables S4–S6 in the Supplementary Information.

The DFT-calculated HOMO energy level of compound **3c** is −6.406 eV. The introduction of electron-donating amino (compounds **5a**, **5b**, and **5g**) and o-tolylthio (**5e**) substituents results in an increase in the HOMO energy to −5.573 eV, −5.706 eV, −5.510 eV, and −5.9049 eV, respectively. The opposite effect is observed for substituents containing bromine (**3h**), nitro (**3i**), and 1,2,4-triazol-1-yl groups (**5c** and **5f**), which lead to a decrease in the HOMO energy to −6.7212 eV, −6.9525 eV, −6.5552 eV, and −6.9307 eV, respectively.

Inductive effects involving changes in molecular polarization caused by electron-withdrawing and electron-donating groups also influence the LUMO energy values. For compound **3c**, the LUMO energy value is 2.3402 eV. Amino groups, mainly acting as electron donors through a strong mesomeric effect (+M), increase the LUMO levels to −2.122 eV, −2.101 eV, and −1.9429 eV for compounds **5a**, **5b**, and **5g**, respectively. The o-tolylthio group (**5e**), mainly acting as an electron donor through the mesomeric effect (+M), also leads to an increase in the LUMO level to −2.2585 eV.

The opposite effect is observed for compounds with bromine (**3h**) and nitro (**3i**), with values equal to −2.7892 eV and −3.2409 eV, respectively. The bromine substituent, due to its strong negative inductive effect (−I) and its weaker mesomeric effect (+M), leads to an increase in the LUMO energy. An even stronger effect is observed for the nitro group, for which both the inductive (−I) and mesomeric (−M) effects are negative, resulting in an even more pronounced elevation in the LUMO energy. A lowering of the LUMO energy is also observed for the compound containing a 1,2,4-triazol-1-yl substituent (**5c**), with the value reaching −2.6449 eV. This effect is associated with electron withdrawal by the triazole ring, which acts as a strong electron-withdrawing heteroaromatic system. The lowering of the LUMO level becomes even more pronounced when an additional electron-withdrawing nitro substituent is introduced into molecule **5f**. The enhancement of the overall electron-deficient character of the system leads to a LUMO energy of −3.0177 eV.

These results clearly indicate that substitution of the naphthalene core significantly affects frontier orbital energies. Different electron-donating amino groups increase both HOMO and LUMO energy levels, while the electron-withdrawing group induces a slight decrease in these energy levels. Notably, both types of substituents lead to a reduction in the HOMO–LUMO gap compared to the reference compound, with the electron-donating group producing a more pronounced narrowing of the gap than the electron-withdrawing group. Additionally, where mesomeric and inductive effects are combined, the modification is greater, as indicated by the results for compounds **3i** and **5f**.

### 2.3. Optical Properties

**Figure 2:**
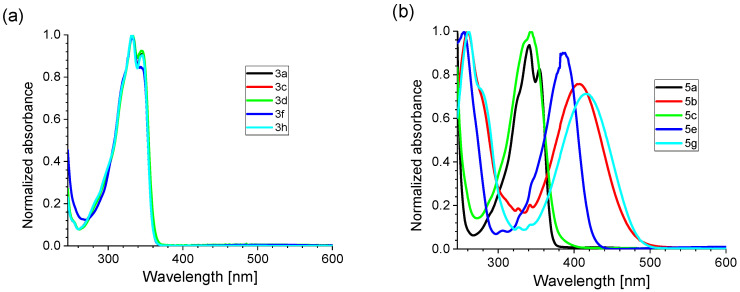
UV–Vis spectra of the selected studied compounds in acetonitrile solution: (**a**) NI derivatives with different N-hydroxyalkyl chains (**3a**, **3c**, **3d**, **3f**, **3h**), and (**b**) NI derivatives substituted on the naphthalene ring (**5a**, **5b**, **5c**, **5e**, **5g**).

The optical properties of the studied compounds were investigated using UV–Vis fluorescence spectroscopy. Measurements were conducted in toluene and acetonitrile (ACN) solutions. The normalized UV–Vis spectra of selected compounds are presented in Figure 2, while the corresponding photophysical parameters are summarized in Table 2. Spectra for different concentrations of all compounds are presented in the Supplementary Information.

The absorption spectra of the compounds exhibit complex features, primarily due to the influence of the condensed naphthalene ring. Significant variations in absorption maxima *λ_abs,_* ranging from 330 nm to 415 nm in ACN and from 332 nm to 410 nm in toluene, are observed. The results indicate a slight solvatochromic effect when these two solvents with different polarities are used. In the case of NI derivatives **3a**–**3g**, with an unsubstituted naphthalene core, only minor changes are observed in both the spectral shape and absorption maxima, likely attributed to steric effects.

**Figure 3:**
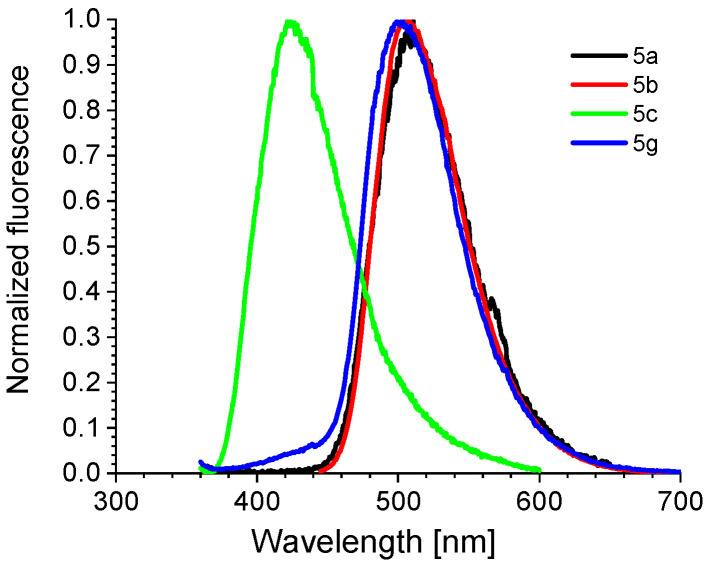
Fluorescence spectra of the selected compounds (**5a**, **5b**, **5c**, **5g**) recorded in toluene solution.

In contrast, a bathochromic shift is observed for compounds **3i** and **5a**–**g**. The substitution of the naphthalene ring with various functional groups introduces diverse mesomeric and inductive effects. Even structurally similar amine substituents can have clearly different electronic effects on the UV–Vis absorption profiles. As an illustrative example, compounds **5a** and **5b** display substantially divergent spectral characteristics. Nevertheless, detailed spectral analysis reveals that compound **5a** also exhibits absorption features above 400 nm, although the corresponding bands are significantly less intense compared to those observed for compound **5b**. Such differences can be attributed to the distinct steric profiles of these substituents, which impose different spatial orientations within the molecular framework. As demonstrated in previous studies, variations in the conformational preferences of NI units arise primarily from steric hindrance introduced by adjacent hydrogen atoms [42]. Subsequent fluorescence measurements substantiate this hypothesis, as the emission spectra reveal comparable energies of the emission maxima of **5a** and **5b**, as shown in Figure 3. This indicates a similar structure in the excited state.

Fluorescence properties also vary significantly among the compounds. Among **3a**–**3e**, a strong dependence on the branching of the hydroxyalkyl groups is evident. Compounds with **3a**–**3c** as linear substituents exhibit low QY, typically not exceeding 7%. However, for compounds **3d** and **3e**, the QY increases to 19%. This enhancement is likely due to intramolecular hydrogen bonding between carbonyl and the two hydroxyl groups, which rigidifies the molecular structure, reduces non-radiative vibrational relaxation, and promotes radiative decay from the excited state to the ground state. The effect of hydrogen bonding has also been reported for different groups of compounds [43,44,45,46].

Similarly, substantial differences in fluorescence efficiency are observed among the second series of naphthalene-substituted compounds. These variations are closely linked to the nature of the substituent. In the case of compounds **5a**, **5b**, **5c** and **5g**, a significant increase in QY is observed relative to **3c**. It is indicated that for naphthaleneimides, a significant increase in QY is associated with the formation of an exciplex between the naphthalimide ring and the amine and triazolyl substituents, which is stabilized by electron transfer [47]. In contrast, the NO_2_ group reduces the QY both in **3i** and **5f**. In compound **3i**, the NO_2_ group is directly attached to the naphthalene core, whereas in compound **5f**, it is connected through a triazole moiety. The quenching effect of NO_2_ groups is attributed to their non-radiative deactivation related to strong electron-withdrawing properties [48].

The optical gap values estimated from measurements in acetonitrile ${Eg}_{ACN}^{opt}$ and toluene ${Eg}_{toluene}^{opt},$ determined from the wavelength corresponding to the beginning of light absorption, were also compared (Table 3). For the **3a**–**3f** series, clear but slight trends were identified. Compounds **3d** and **3e**, with branched hydroxyalkyl groups, have slightly lower ${Eg}_{ACN}^{opt}$ and ${Eg}_{toluene}^{opt}$ than compounds **3a**–**3c**, with linear hydroxyalkyl groups. This is due to the minor steric effects of their more extensive branched side chains.

In the case of substitution of the naphthalene core by substituents such as amines, nitro groups, o-tolylthio groups or 1,2,4-triazoyl, we observed a decrease in the value of ${Eg}_{ACN}^{opt}$ and ${Eg}_{toluene}^{opt}$, even though these groups are characterized by different positive and negative mesomeric effects. The trend is largely consistent with the DFT-calculated energy differences in the HOMO and LUMO levels ${Eg}_{HOMO-LUMO}^{DFT}$. In all cases, the introduction of substitutions results in a reduction in the various types of energy gaps.

### 2.4. Electrochemistry

**Figure 4:**
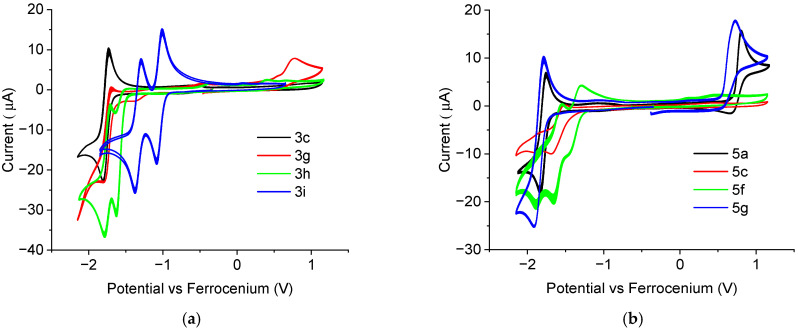
Cyclic Voltammetry of the selected compounds in dichloromethane/Bu_4_NPF_6_ as electrolyte: (**a**) **3c**, **3g**, **3h**, **3i** and (**b**) **5a**, **5c**, **5f**, **5g**.

The studied compounds were subjected to electrochemical measurements using cyclic voltammetry. The measurements were performed in a solution of dichloromethane and tetrabutylammonium hexafluorophoshate (Bu_4_NPF_6_) as an electrolyte. Reduction peaks were registered for all compounds. Oxidation peaks were recorded whenever they occurred within the potential windows of the electrolytes used. The values determined for the redox potentials and oxidation and reduction onset potentials estimated vs. the ferrocene/ferrocenium redox couple are collected in Table 4. For clarity of presentation, the cyclic voltammetry (CV) of selected naphthaleneimide derivatives is shown in Figure 4. CVs of all compounds are collected in Supplementary Materials.

Detailed analysis of the CVs shows clear trends and differences depending on the structure of the studied compounds. In the case of the **3a**–**3f** NI derivatives, the reduction potential is similar for all compounds. The slight differences may be due to the steric effects of substituents on the nitrogen atom. Unsubstituted naphthalimides are typical aromatic compounds that are readily reduced but much more difficult to oxidize. This is related to the presence of the strong electron-withdrawing effects of the imide group, and hence the presence of an electron-deficient NI core [29].

An oxidation peak is observed for compound **3g**. However, this observation, in combination with other techniques, indicates that this is not the direct oxidation of **3g** molecules. The probable cause of this oxidation peak is the formation of π-stacks of **3g** at the electrode surface. In the case of **3a**–**3f**, the formation of such interactions is not observed. This indicates the blocking of π-stacks by the hydroxyl group.

For derivatives **3h**, **3i** and **5a**–**5g**, the electrochemical properties are influenced by the substitution of the naphthalene ring with various types of substituents. The change in their electrochemical properties, including oxidation and reduction potentials, is influenced by substituents that change the electron density of naphthalene rings through mesomeric and inductive effects. In the case of **5a**, **5b** and **5g** compounds, different amine groups decrease their reduction potential because of their electron-donating effect, which causes an increase in electron density on the naphthalene ring. The opposite effect is observed for **3h**, **3i**, **5c**, **5e** and **5f** compounds. A particularly strong effect is observed for **3i**. The nitro group increases the reduction potential to a value of −0.99 V. The reversibility of two reduction peaks is also observed for this derivative. This is the result of combining the negative mesomeric and the electron-withdrawing inductive effects, which cause a decrease in the electron density on the naphthalene ring.

The reduction potential of chemical molecules is related to the energy of their LUMO orbital. The oxidation potential of molecules, in turn, is related to the energy of their HOMO orbital. Therefore, these values can be compared. There are clear trends between the calculated LUMO energy values and the reduction potentials. For compounds in which substituents exhibit a decrease in the LUMO orbital energy value, an increase in oxidation potential occurs. Only in the case of compound **5e** do we observe a slight deviation from this trend. In turn, for compounds for which an increase in the HOMO potential was calculated, their oxidation peak is observed at a relatively low potential.

### 2.5. Batteries

The studied series of naphthalene imide derivatives exhibits broad potential for application, leveraging both their optical and electrochemical properties. Several compounds demonstrated promising electrochemical behavior. To explore their practical applicability, a few test batteries incorporating these compounds were fabricated.

Selected compounds were evaluated for their potential use as cathode materials in lithium-ion cells. For this purpose, a set of 2032-type test coin cells was assembled. In these cells, pure lithium chips served as the anode, a glass microfiber membrane was used as the separator, and the electrolyte consisted of a 1 M solution of LiPF_6_ in a 1:1:1 volume ratio mixture of ethylene carbonate (EC), diethyl carbonate (DEC), and ethyl methyl carbonate (EMC). The cathodes were prepared using the spin coating method.

The assembled batteries were tested under an argon atmosphere using the galvanostatic charge–discharge (GCD) method within a voltage range of 3.0 to 4.0 V and at various current densities ranging from 2 to 0.03 A/g. Chosen results are summarized in Table 5.

**Figure 5:**
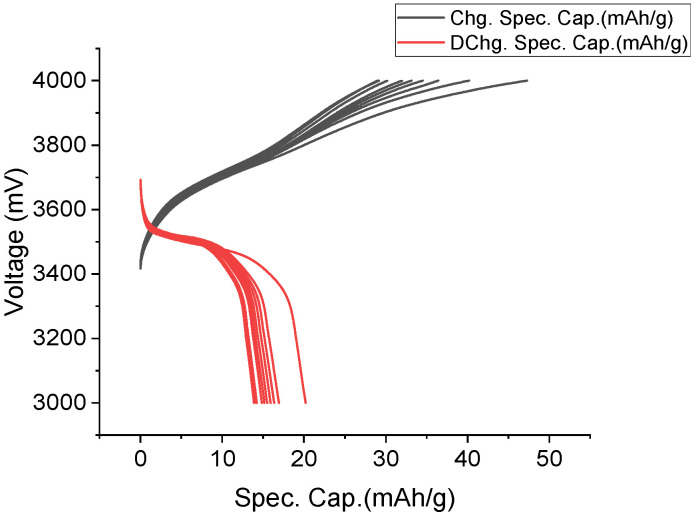
GCD plot registered for the battery with **3i** as a cathode material with applied 50 μA current, which corresponds to app 0.15 A/g.

**Figure 6:**
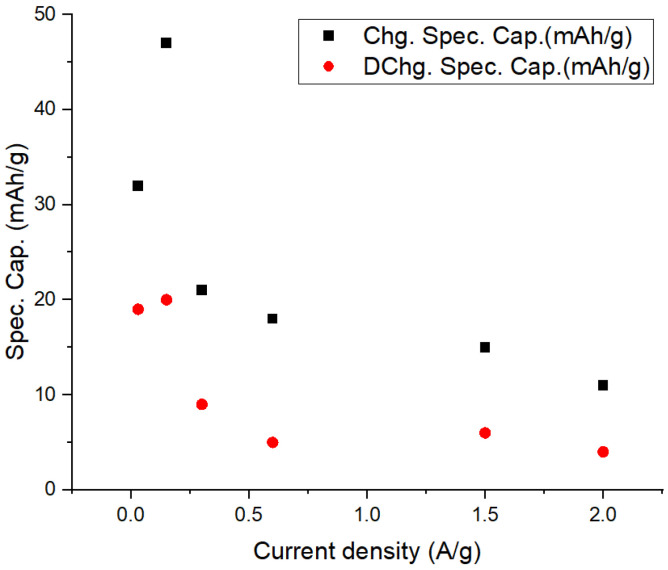
Rate capability of the battery (**3i**)—specific capacity of tested battery vs. current density applied.

The best performance was observed for the test battery using compound **3i** as the cathode material (Figure 5 and Figure 6). With this setup, we achieved specific discharge capacities of 19, 20, 9, 5, 6 and 4 mAh/g at current densities of 30, 150, 300, 600, 1500 and 2000 mA/g, respectively. With increased current density, the specific capacity gradually decreased. It seems that the best performance was obtained for a current density of 0.15 A/g. The initial reversible charge/discharge capacities were approximately 47/20 mAh/g, which gradually decreased initially but later stabilized at around 30/14 mAh/g over multiple cycles (stable behavior after 60 charging/discharging cycles). Throughout the testing period, the charge/discharge efficiency remained at approximately 50%. Other materials yielded similar but slightly lower performance. Comparing the results obtained here with literature data, we obtain approximately 25–30% of the capacity of typical lithium-ion cathodes (120–250 mAh/g) [49,50].

## 3. Experiment

### 3.1. Materials and Methods

All reagents were purchased from commercially available sources (Merck, Sigma-Aldrich Darmstadt, Germany; Avantor Performance Materials, Gliwice, Poland). Thin-layer chromatography plates (60F254) and Silica Gel 60 (0.040–0.063 mm) for column chromatography were purchased from Merck (Darmstadt, Germany). Visualization of the spots on TLC plates was achieved by UV light. Product purification was performed using the Chromatography system LaboACE LC-5060 (Japan Analytical Industry Co., Ltd, Tokio, Japan). The structure of the synthesized compounds was analyzed with NMR at 600 MHz for ^1^H NMR and 150 MHz for ^13^C NMR; δ values are in parts per million (ppm) relative to tetramethylsilane (TMS) as an internal standard or to the solvent residual peak. High-resolution electrospray ionization mass spectroscopy (ESI-MS) experiments were performed using a Waters Xevo G2 QTOF instrument (Milford, MA, USA) equipped with an injection system (cone voltage 50 V; source 120 °C). Melting points were determined using a digital melting point apparatus (Electrothermal IA9100, Bionovo, Legnica, Poland).

The starting 1,8-naphthalic anhydrides **1b** and **1c** were obtained from acenaphthene according to the reported procedure [36]. **1b** was received in 63% yield, covering both steps of bromination (83%) and oxidation (76%). 4-Nitro-1,8-naphthalic anhydride 1c was obtained in 46% total yield (comprising nitration and oxidation). Unsubstituted 1,8-naphthalic anhydride **1a** was purchased from Merck.

2-(ω-Hydroxyalkyl)-1*H*-benzo[*de*]isoquinoline-1,3(2*H*)-diones **3a**–**3f**, **3h**–**3i** general procedure

To a solution of 1,8-naphthalic anhydride **1a**–**1c** (2 mmol) in ethanol (15 mL), an equimolar amount of the appropriate amino alcohol, **2a**–**f** (2 mmol), was added. Then the mixture was refluxed for 4 h and cooled to room temperature. After chilling, the precipitated solid was filtered off, washed thoroughly with distilled water, and recrystallized from absolute ethanol to yield a highly pure product.

Under the same conditions, derivative **3g** was prepared by treating the anhydride **1a** with 1,4-butylenediamine **2g**.

2-(4-Hydroxybutyl)-1*H*-benzo[*de*]isoquinoline-1,3(2*H*)-dione (**3a**)
White solid, Yield 89%. Mp:108–109 °C. ^1^H NMR (CDCl_3_, 600 MHz): δ 8.60 (dd, *J* = 1.2 Hz, 7.2 Hz, 2H), 8.22 (dd, *J* = 1.2 Hz, 8.4 Hz, 2H), 7.76 (dd, *J* = 7.2 Hz, 8.4 Hz, 2H), 4.24 (t, *J* = 7.2 Hz, 1H), 3.75 (t, *J* = 6.6 Hz, 2H), 1.85(p, *J* = 7.2 Hz, 2H), 1.73–1.64 (m, 2H). ^13^C NMR (CDCl_3_, 150 MHz): δ 164.2, 133.9, 131.5, 131.2, 132.2, 128.1, 126.9, 122.3, 62.5, 39.9, 29.9, 24.5. C_16_H_15_NO_3_. ESI-MS (*m*/*z*): 270.1052 [M + H]^+^.
2-(5-Hydroxypentyl)-1*H*-benzo[*de*]isoquinoline-1,3(2*H*)-dione (**3b**)
White solid, Yield 87%. Mp:102–104 °C. ^1^H NMR (DMSO-d_6_, 600 MHz): δ 8.47 (dd, *J* = 1.2 Hz, 7.2 Hz, 2H), 8.42 (dd, *J* = 1.2 Hz, 8.4 Hz, 2H), 7.85 (dd, *J* = 7.2 Hz, 8.4 Hz, 2H), 4.35 (t, *J* = 5.4 Hz, 1H), 4.03 (t, *J* = 7.2 Hz, 2H), 3.41 (q, *J* = 5.4 Hz, 2H), 1.64 (p, *J* = 7.8 Hz, 2H), 1.48 (p, *J* = 7.8 Hz, 2H), 1.40–1.35 (m, 2H). ^13^C NMR (DMSO-d_6_, 150 MHz): δ 163.2, 134.1, 131.2, 130.6, 127.2, 127.1, 121.9, 60.4, 32.2, 27.3, 23.1. C_17_H_17_NO_3_. ESI-MS (*m*/*z*): 284.1208 [M + H]^+^.
2-(6-Hydroxyhexyl)-1*H*-benzo[*de*]isoquinoline-1,3(2*H*)-dione (**3c**)
Yellow solid. Yield 84%. Mp: 92–94 °C. ^1^H NMR (CDCl_3_, 600 MHz): δ 8.60 (dd, *J* = 1.2 Hz, 7.2 Hz, 2H), 8.21 (dd, *J* = 1.2 Hz, 8.4 Hz, 2H), 7.75 (dd, *J* = 7.2 Hz, 8.4 Hz, 2H), 4.20–4.19 (m, 2H), 3.65 (t, *J* = 6.6 Hz, 2H), 3.39–3.36 (m, 2H), 1.77 (p, *J* = 7.2 Hz, 2H), 1.61–1.59 (m, 2H), 1.47–1.45 (m, 4H). ^13^C NMR (CDCl_3_, 150 MHz): δ 162.8, 162.1, 149.0, 131.6, 130.0, 129.6, 128.6, 128.3, 126.4, 124.2, 122.7, 122.6, 60.6, 40.2, 32.4, 26.4, 25.2. C_18_H_19_NO_3_. ESI-MS (*m*/*z*): 298.3484 [M + H]^+^.
2-(2,3-Dihydroxypropyl)-1*H*-benzo[*de*]isoquinoline-1,3(2*H*)-dione (**3d**)
Yield%. Mp: 153–154 °C. ^1^H NMR (DMSO-d_6_, 600 MHz): δ 8.46 (dd, *J* = 0.6 Hz, *J* = 7.2 Hz, 2H), 8.42 (dd, *J* = 0.6 Hz, 8.4 Hz, 2H), 7.84 (dd, *J* = 7.2 Hz, 8.4 Hz, 2H), 4.78 (d, *J* = 5.4 Hz, 1H, OH), 4.58 (t, *J* = 6.0 Hz, 1H, OH), 4.22 (dd, *J* = 8.4 Hz, 13.2 Hz, 1H), 3.98 (dd, *J* = 4.8 Hz, 13.2 Hz, 1H), 3.94–3.89 (m, 1H), 3.44–3.38 (m, 2H). ^13^C NMR (DMSO-d_6_, 150 MHz): δ 164.3 (2×), 134.7, 131.8, 131.1, 127.8, 127.6, 122.8, 69.1, 65.1, 43.8. C_15_H_13_NO_4_. ESI-MS (*m*/*z*): 272.0845 [M + H]^+^.
2-(1,3-Dihydroxypropan-2-yl)-1*H*-benzo[*de*]isoquinoline-1,3(2*H*)-dione (**3e**)
Wight solid Mp: 132–134 °C Yield 92%.^1^H NMR (DMSO-d_6_, 600 MHz): δ 8.48 (d, *J* = 7.2 Hz, 2H), 8.44 (dd, *J* = 1.2 Hz, 7.8 Hz, 2 H), 7.84 (dd, *J* = 7.2 Hz, 7.8 Hz, 2H), 5.27–5.23 (m, 1H), 4.79 (dd, *J* = 5.4 Hz, 6.6 Hz, 2H), 3.98–3.94 (m, 2H), 3.83 (dt, *J* = 6.0 Hz, 2H). ^13^C NMR (DMSO-d_6_, 150 MHz): δ 164.8 (2×), 134.3, 131.6, 131.0, 128.0, 127.6, 123.1, 59.4, 58.6. C_15_H_13_NO_4_. ESI-MS (*m*/*z*): 272.0845 [M + H]^+^.
2-(4-(2-hydroxyethyl)phenyl)-1*H*-benzo[*de*]isoquinoline-1,3(2*H*)-dione (**3f**)
White solid. Yield 78%. Mp: 192–194° ^1^H NMR (DMSO-d_6_, 600 MHz): 8.45 (dd, *J* = 1.2 Hz, 3.0 Hz, 2H). 8.44 (dd, *J* = 1.2 Hz, 3.0 Hz, 2H), 7.88 (dd, *J* = 7.2 Hz, 7.8 Hz, 2H), 7.38–7.36 (m, 2H), 7.28–7.26 (m, 2H), 4.78 (t, *J* = 4.8 Hz, 1H), 3.70 (dt, *J* = 5.4 Hz, 6.6 Hz, 2H), 2.83 (t, *J* = 6.6 Hz, 2H). ^13^C NMR (DMSO-d_6_, 150 MHz): δ 163.6, 139.6, 134.3, 133.8, 131.3, 130.7, 129.3, 128.7, 127.7, 127.1, 122.5, 62.0, 38.7. C_20_H_15_NO_3_. ESI-MS (*m*/*z*): 318.1052 [M + H]^+^.
2,2′-(Butane-1,4-diyl)bis(1*H*-benzo[*de*]isoquinoline-1,3(2*H*)-dione) (**3g**)
Yield 87%. Mp: 198–200 °C. ^1^H NMR (CDCl_3_, 600 MHz): δ 8.58 (dd, *J* = 1.2 Hz, 7.8 Hz, 4H), 8.21 (dd, *J* = 1.2 Hz, 8.4 Hz, 4H), 7.73 (dd, *J* = 7.8 Hz, 8.4 Hz, 4H), 4.28–4.25 (m, 4H), 1.90–1.88 (m, 4H). ^13^C NMR (CDCl_3_, 150 MHz): δ 164.2, 133.8, 131.5, 131.1, 128.1, 126.8, 122.6, 40.0, 25.9. C_28_H_20_N_2_O_4_. ESI-MS (*m*/*z*): 449.1423 [M + H]^+^.
6-Bromo-2-(6-hydroxyhexyl)-1*H*-benzo[*de*]isoquinoline-1,3(2*H*)-dione (**3h**)
Yellow solid Yield 85%. Mp: 108–110 °C (lit. 91–92 °C) [36]. ^1^H NMR (DMSO-d_6_, 600 MHz): 8.50 (dd, *J* = 1.2 Hz, 7.2 Hz, 1H). 8.46 (dd, *J* = 1.2 Hz, 8.4 Hz, 1H), 8.25 (d, *J* = 7.8 Hz, 1H), 8.15 (d, *J* = 7.8 Hz, 1H), 7.94 (dd, *J* = 7.2 Hz, 8.4 Hz, 1H), 4.34 (t, *J* = 5.4 Hz, 1H), 3.99 (t, *J* = 7.8 Hz, 2H), 3.38 (dd, *J* = 4.8 Hz, 6.6 Hz, 2H), 1.61 (p, *J* = 7.2 Hz, 2H), 1.42 (p, *J* = 6.6 Hz, 2H), δ 1.33 (p, *J* = 3.6 Hz, 4H), ^13^C NMR (DMSO-d_6_, 150 MHz): δ 163.7, 163.6, 133.4, 132.4, 132.2, 131.8, 130.6, 129.9, 129.6, 129.1, 123.6, 122.8, 61.5, 40.7, 33.3, 28.4, 27.3, 26.1. C_18_H_18_BrNO_3_. ESI-MS (*m*/*z*): 377.2444 [M + H]^+^, 378.0450 (M + 2 + H]^+^.
2-(6-Hydroxyhexyl)-6-nitro-1*H*-benzo[*de*]isoquinoline-1,3(2*H*)-dione (**3i**)
Yellow solid. Yield 87%. Mp: 110–112 °C. (lit. Mp 112–113 °C) [36] ^1^H NMR (CDCl_3_, 600 MHz): δ 8.84 (d, *J* = 9.0 Hz, 1H), 8.74 (d, *J* = 7.2 Hz, 1H), 8.69 (d, *J* = 7.8 Hz, 1H), 8.40 (d, *J* = 8.4 Hz, 1H), 7.99 (dd, *J* = 7.8 Hz, 9.0 Hz, 1H), 4.21 (t, *J* = 7.8 Hz, 2H), 3.68 (t, *J* = 6.6 Hz, 2H), 3.38 (t, *J* = 7.6 Hz, 2H), 1.79 (p, *J* = 7.8 Hz, 2H), 1.67 (p, *J* = 6.6 Hz, 2H), 1.54–1.49 (m, 2H). ^13^C NMR (CDCl_3_, 150 MHz): δ 163.7, 162.8, 148.6, 132.8, 130.3, 130.2, 129.7, 127.3, 124.2, 63.7, 41.0, 32.7, 28.1, 23.6. C_18_H_18_N_2_O_5_. ESI-MS (*m*/*z*): 343.1216 [M + H]^+^.
2-(ω-Hydroxyalkyl)-6-substituted-1*H*-benzo[*de*]isoquinoline-1,3(2*H*)-diones **5a**–**g**
A mixture of compound **3h** (1 mmol) and an acyclic or heterocyclic amine (1.2 mmol) was dissolved in anhydrous DMF (5–10 mL) containing triethylamine (Et_3_N, 0.06 mL, 0.5 mmol) as the base. The reaction mixture was stirred and heated at 90 °C for approximately 2–4 h. The reaction’s progress was monitored by TLC using a solvent system (MeOH:CHCl_3_, 2:10, *v*/*v*). Once the reaction was completed, the mixture was poured into water (50 mL). A series of three liquid–liquid extractions was then performed using 50 mL of water and 30 mL of chloroform for each extraction to remove DMF, allowing the target compound, which is soluble in chloroform, to separate. The combined organic layer was washed with a saturated solution of NaHCO_3_, dried over Na_2_SO_4_ and filtered. After solvent evaporation, the residue was purified by crystallization using a mixture of anhydrous ethanol and diethyl ether.
2-(6-Hydroxyhexyl)-6-morpholino-1*H*-benzo[*de*]isoquinoline-1,3(2*H*)-dione (**5a**)
Yellow solid, yield 88%. Mp: 123–124 °C. ^1^H NMR (DMSO-d_6_, 600 MHz): δ 8.47 (dt, *J* = 1.2 Hz, 8.4 Hz, 2H), 8.39 (d, *J* = 8.4 Hz, 1H), 7.80 (dd, *J* = 7.2 Hz, 8.4 Hz, 1H), 7.34 (d, *J* = 8.4 Hz, 1H), 4.34 (t, *J* = 5.4 Hz, 1H), 4.03–4.01 (m, 2H), 3.91 (t, *J* = 4.2 Hz, 4H), 3.39–3.36 (m, 2H), 3.21 (t, *J* = 4.2 Hz, 4H), 1.60 (p, *J* = 6.6 Hz, 2H), 1.41 (p, *J* = 6.6 Hz, 2H) 1.34–1.31 (m, 4H). ^13^C NMR (DMSO-d_6_, 150 MHz): δ 164.4, 163.9, 156.3, 133.0, 131.6, 131.4, 127.0, 126.2, 123.5, 116.8, 115.9, 67.1, 61.6, 53.9, 40.4, 33.4, 28.5, 27.4, 26.2. C_22_H_26_N_2_O_4_. ESI-MS (*m*/*z*): 383.1893 [M + H]^+^.
2-(6-Hydroxyhexyl)-6-(piperidin-1-yl)-1*H*-benzo[*de*]isoquinoline-1,3(2*H*)-dione (**5b**)
Yield 78%. Mp: 116–118 °C (lit. 133–133.2 °C [38]). ^1^H NMR (CDCl_3_, 600 MHz): δ 8.49 (dd, *J* = 1.2 Hz, 7.2 Hz, 1H), 8.41 (d, *J* = 8.4 Hz, 1 H), 8.30 (dd, *J* = 1.2 Hz, 8.4 Hz, 1H), 7.59 (t, *J* = 8.40 Hz, 1H), 7.09 (d, *J* = 8.4 Hz, 1H), 4.10 (t, *J* = 7.2 Hz, 2H), 3.56 (t, *J* = 6.0 Hz, 2H), 3.18–3.13 (m, 4H), 1.81 (p, *J* = 5.4 Hz, 4H), 1.70–1.63 (m, 4H), 1.52 (p, *J* = 6.6 Hz, 2H), 1.41–1.38 (m, 4H). ^13^C NMR (CDCl_3_, 150 MHz): δ 165.1, 164.4, 133.1, 131.4, 131.0, 130.3, 126.6, 125.7, 123.5, 116.3, 115.1, 63.1, 54.9, 40.3, 32.9, 28.4, 27.0, 26.6, 25.6, 24.7. C_23_H_28_N_2_O_3_. ESI-MS (*m*/*z*): 381.2133 [M + H]^+^.
2-(6-Hydroxyhexyl)-6-(1*H*-1,2,4-triazol-1-yl)-1*H*-benzo[*de*]isoquinoline-1,3(2*H*)-dione (**5c**)
Yellow solid, yield 85%. Mp: 196–198 °C. ^1^H NMR (DMSO-d_6_, 600 MHz): δ 9.24 (s, 1H), 8.58 (t, *J* = 7.2 Hz, 2H), 8.48 (s, 1 H), 8.35 (d, *J* = 8.4 Hz, 1H), 8.07 (d, *J* = 7.8 Hz, 1H), 7.92 (dd, *J* = 7.8 Hz, 8.4 Hz, 1H), 4.35 (t, *J* = 5.4 Hz, 1 H), 4.04 (t, *J* = 7.8 Hz, 2H), 3.39 (dd, *J* = 6.6 Hz, 12 Hz, 2H), 1.64 (p, *J* = 7.2 Hz, 2H), 1.43 (p, *J* = 6.6 Hz, 2H), 1.40–1.35 (m, 4H). ^13^C NMR (DMSO-d_6_, 150 MHz): δ 164.1, 163.6, 154.0, 147.3, 138.8, 132.2, 131.4, 130.5, 129.4, 129.3, 126.6, 124.8, 123.8, 123.4, 61.6, 40.8, 33.4, 28.5, 27.3, 26.2. C_20_H_20_N_4_O_3_. ESI-MS (*m*/*z*): 365.1569 [M + H]^+^.
6-(Diethylamino)-2-(6-hydroxyhexyl)-1*H*-benzo[*de*]isoquinoline-1,3(2*H*)-dione (**5d**)
Yield 75%. Mp: 112–113 °C ^1^H NMR (CDCl_3_, 600 MHz): δ 8.64 (dd, *J* = 1.2 Hz, 7.8 Hz, 1H), 8.55 (dd, *J* = 1.2 Hz, 8.4 Hz, 1H), 8.40 (d, *J* = 7.8 Hz, 1H), 8.02 (d, *J* = 7.8 Hz, 1H), 7.83 (dd, *J* = 7.2 Hz, 8.4 Hz, 1H), 4.18–4.15 (m, 2H), 3.64 (t, *J* = 6.6 Hz, 2H), 1.75 (p, *J* = 7.2 Hz, 2H), 1.59 (p, *J* = 7.2 Hz, 2H), 1.46–1.44 (m, 4H).^13^C NMR (CDCl_3_,150 MHz): δ 164.0, 163.9, 133.6, 132.4, 131.6, 131.4, 131.0, 130.6, 129.3, 128.4, 123.4, 122.6, 63.1, 40.7, 33.0, 28.3, 27.1, 25.7. C_22_H_28_N_2_O_3_. ESI-MS (*m*/*z*): 369.2133 [M + H]^+^.
2-(6-Hydroxyhexyl)-6-(*o*-tolylthio)-1*H*-benzo[*de*]isoquinoline-1,3(2*H*)-dione (**5e**)
Green-yellow solid, yield 82%. Mp: 174–175 °C. ^1^H NMR (CDCl_3_, 600 MHz): δ 8.66–8.63 (m, 2H), 8.31 (d, *J* = 7.8 Hz, 1 H), 7.79 (dd, *J* = 7.2 Hz, 8.4 Hz, 1H), 7.53 (d, *J* = 7.2 Hz, 1H), 7.43–7.7.40 (m, 2H), 7.31–7.27 (m, 1H), 6.98 (d, *J* = 7.8 Hz, 1H), 4.17–4.15 (m, 2H), 3.64 (t, *J* = 6.6 Hz, 2H), 2.40 (s, 3H), 1.74 (p, *J* = 7.2 Hz, 2H), 1.59 (p, *J* = 7.2 Hz, 2H), 1.46–1.43 (m, 4H). ^13^C NMR (CDCl_3_, 150 MHz): δ 164.4, 164.3, 145.5, 142.8, 136.5, 131.9, 131.8, 131.2, 130.6, 129.4, 129.3, 128.8, 127.9, 123.5, 119.9, 63.1, 40.5, 32.9, 28.3, 27.1, 25.6, 20.9. C_25_H_25_NO_3_S. ESI-MS (*m*/*z*): 420.1633 [M + H]^+^.
2-(6-Hydroxyhexyl)-6-(3-nitro-1*H*-1,2,4-triazol-1-yl)-1*H*-benzo[*de*]isoquinoline-1,3(2*H*)-dione (**5f**)
Red solid, yield 70%. Mp: 184–185 °C. ^1^H NMR (CDCl_3_, 600 MHz): δ 8.74 (dd, *J* = 1.2 Hz, 7.2, Hz, 1H), 8.73 (d, *J* = 7.8 Hz, 1H), 8.67 (s, 1H), 8.24 (dd, *J* = 1.2 Hz, 8.4 Hz, 1H), 7.93 (dd, *J* = 7.2 Hz, 8.4 Hz, 2H), 4.20 (dt, *J* = 1.2 Hz, 7.2 Hz, 4H), 3.65 (t, *J* = 6.6 Hz, 1H), 1.80–1.75 (m, 2H), 1.61 (p, *J* = 6.6 Hz, 2H), 1.48–1.46 (m, 4H). ^13^C NMR (CDCl_3_, 150 MHz): δ 163.9, 163.4, 149.6, 149.5, 137.3, 132.5, 131.1, 130.0, 129.9, 129.1, 126.6, 126.1, 125.1, 123.5, 61.5, 40.9, 33.3, 28.4, 27.3, 26.2. C_20_H_19_N_5_O_5_. ESI-MS (*m*/*z*): 410.1420 [M + H]^+^.
2-(6-Hydroxyhexyl)-6-(6-hydroxyhexylamino)-1*H*-benzo[*de*]isoquinoline-1,3(*2H*)-dione (**5g**)
Yellow solid. Yield 88%. Mp: 86–88 °C. ^1^H NMR (CDCl_3_, 600 MHz): δ 8.56 (dd, *J* = 1.2 Hz, 8.4 Hz, 1H), 8.47 (dd, *J* = 1.2 Hz, 8.4 Hz, 1H), 8.43(dd, *J* = 1.2 Hz, 8.4 Hz, 1H), 7.65 (ddd, *J* = 1.2 Hz, 7.2 Hz, 8.4 Hz, 1H), 7.12 (dd, *J* = 1.2 Hz, 8.4 Hz, 1H), 4.17 (t, *J* = 7.8 Hz, 2H), 3.64 (t, *J* = 6.6 Hz, 2H), 3.10 (s, 6H), 3.08 (s, 6H), 1.77–1.72 (m, 2H), 1.50–1.41 (m, 4H). ^13^C NMR (CDCl_3_, 150 MHz): δ 165.1, 164.5, 157.3, 132.9, 131.4, 131.3, 130.5, 125.6, 125.2, 123.4, 115.3, 113.6, 60.1, 60.5, 45.1, 40.3, 32.9, 29.9, 28.3, 26.9, 25.6. C_24_H_32_N_2_O_4_. ESI-MS (*m*/*z*): 413.2396 [M + H]^+^.

### 3.2. Optical Measurement

Fluorescence measurements were carried out using a Modular Fluorescence Spectrometer fluoroSENS Pro11 (Camlin, Lisburn, Ireland). UV–Vis measurements were carried out using an HP 8453 spectrometer. Absorption spectra and fluorescence spectra of the studied compounds were recorded in toluene solutions with a concentration of 2 × 10^−5^ mol∙dm^−3^. Photoluminescence quantum yield Φ_f_ of **5b**, **5c** and **5h** were determined by the direct measurement method using an integrating sphere. In the case of derivatives that were characterized by weaker emissions, they were determined using an indirect method. In this method, a larger optical slit can be used for the excitation and emission beams. The procedure was estimated in accordance with the methodology outlined in [51], employing **5c** as the reference standard.

### 3.3. Electrochemical Measurement

Electrochemical measurements were performed in a three-electrode electrochemical cell, employing a platinum wire (Pt) as the working electrode, a platinum spiral as the counter electrode, and an Ag/AgCl reference electrode model ET072 (eDAQ, Denistone East, Australia). The experiments were conducted using a CH Instruments Electrochemical Workstation Model CHI660C (CH Instruments, Inc., Austin, TX, USA). Compounds with a concentration of 2 × 10^−3^ mol∙dm^−3^ in an acetonitrile/Bu_4_NPF_6_ electrolyte were used for the measurements. The potential was shifted by the ferrocene/ferrocenium redox couple potential. The smoothing function was used to reduce noise.

### 3.4. DFT Calculations

DFT calculations were performed using the Orca 6.0.1 program package [52,53] with the B3LYP potential [54] and 6-31G(d,p) basis set. The **3h** compound (containing a bromine atom) was used exclusively with the 6-311G(d,p) basis set. All eigenvalues of the Hessians were positive for all compounds subjected to calculations. All results were calculated for the gas phase.

### 3.5. Battery Fabrication

A set of 2032-type test coin cells was prepared. The anode consisted of pure lithium chips (Gelon, Dongguan, China), while a glass microfiber membrane (Whatman 934-AH) was used as the separator. The electrolyte was a 1 M solution of LiPF_6_ in a 1:1:1 volume ratio mixture of ethylene carbonate (EC), diethyl carbonate (DEC), and ethyl methyl carbonate (EMC). The cathode was prepared using the drop casting method with a Spin Coater Model WS-650MZ-23NPP/LITE (Laurell Technologies Corporation, Lansdale, PA, USA). For this, a mixture of studied NI derivative/carbon black/PVDF 1.46/0.72/0.243 mg in 200 µL of DCM was applied to a stainless-steel disk (18 mm diameter, 0.5 mm thick). The coated disk was then heated on a hot plate at 100 °C for 15 min and used immediately. Cell assembly was conducted in a glovebox under an argon atmosphere, with O_2_ and H_2_O concentrations maintained below 0.1 ppm. The assembled batteries were evaluated using the galvanostatic charge–discharge (GCD) method within a voltage range of 3.0 to 4.0 V and at various current densities ranging from 2 to 0.03 A/g. The experiments were conducted using a Neware BTS model CT408T (Dongguan, China).

## 4. Conclusions

We successfully synthesized two series of novel 1,8-naphthalimide derivatives. The first series features various N-hydroxyalkyl substituents at the imide position, while the second comprises compounds with different functional groups attached to the naphthalene core. The nature of the substituents significantly influenced both the electrochemical and optical properties of the synthesized compounds. The UV–Vis spectra varied considerably with molecular structure; most compounds absorbed primarily in the ultraviolet region, although certain modifications led to bathochromic shift into the visible range. Fluorescence properties were also strongly dependent on substitution patterns at both the imide and naphthalene positions, with quantum yields ranging from 0% to 96%. The presence of branched hydroxyalkyl substituents enhanced fluorescence efficiency, whereas the introduction of nitro groups led to significant fluorescence quenching. The reduction potential varied by more than 0.7 V depending on the substituent. The reversibility of the reduction processes also depended on the characteristics of the substituent. The combination of the nitro group’s negative mesomeric effect (-M) and its electron-withdrawing inductive effect (-I) in molecule **3i** proved to be particularly advantageous. This derivative has also shown promise as a cathode material in lithium-ion batteries.

## Figures and Tables

**Table 1:** Reaction optimization conditions for the synthesis of compound **5a** from compound **3h**.

Entry	Catalyst (% mmol)	Solvent (5 mL)	Condition °C	Times (h)	Yields (%)
1	Sans catalyst	CHCl_3_	r.t	24	*
2	Morpholine 0.5 eq	THF	Reflux	12	30
3	4-Ppy (0.03 mL)	EtOH	Reflux	6	*
4	4-Ppy (0.03 mL)	1,4-Dioxane	90	5	50
5	4-Ppy	DMF	100	4	60
6	DBU (0.03 mL)	1,4-Dioxane	reflux	4	80
7	DBU	DMF	90	3	87
8	K_2_CO_3_ (1.5 eq)	DMF	100	4	70
9	Et_3_N (0.5 eq)	DMF	90	2–4	88

* Product not detected.

**Table 2:** Optical properties: absorption spectrum maxima in ACN and toluene (*λ_abs_*); fluorescence emission spectrum maxima in toluene (*λ_em_*); fluorescence quantum yield (QY).

Code	*λ_abs_*	*λ_abs_*	*λ_em_*	QY
	[nm]	[nm]	[nm]	[%]
	ACN	Toluene	Toluene	Toluene
**3a**	330, 340	332, 347	415	4
**3b**	331, 340	332, 348	415	3
**3c**	331, 344	333, 348	415	3
**3d**	332, 344	337	427	19
**3e**	331, 344	335	427	19
**3f**	321, 334	330	*	0
**3g**	330, 341	331, 347	412	5
**3h**	330, 342	341, 357	420	0
**3i**	348	351	*	0
**5a**	339, 352	343	510	96
**5b**	406	397	507	53
**5c**	341	343	424	55
**5e**	394	380, 389	471	1
**5f**	332, 342, 418	341, 410	429	3
**5g**	416	409	500	84

* Not recorded.

**Table 3:** Optical band gaps estimated from the equation ${Eg}_{solvent}^{opt}$ = 1240/λ_abs onset_ in acetonitrile (${Eg}_{ACN}^{opt}$) and toluene $\left({Eg}_{toluene}^{opt}\right)$; electrochemical gap estimated from CV (${Eg}^{el}$); DFT-calculated HOMO–LUMO energy difference (${Eg}_{HOMO-LUMO}^{DFT}$).

Code	${Eg}_{ACN}^{opt}$	${Eg}_{toluene}^{opt}$	${Eg}^{el}$	${Eg}_{HOMO-LUMO}^{DFT}$
	[eV]	[eV]	[eV]	[eV]
**3a**	3.41	3.42	*	
**3b**	3.39	3.41	*	
**3c**	3.41	3.43	*	4.0654
**3d**	3.33	3.40	*	
**3e**	3.32	3.34	*	
**3f**	3.36	3.34	*	
**3g**	3.38	3,40	2.43	
**3h**	3.44	3.32	*	3.932
**3i**	2.62	2.74	*	3.7116
**5a**	2.59	2.69	2.54	3.451
**5b**	2.46	2.67	2.47	3.605
**5c**	3.07	3.22	*	3.9103
**5e**	2.83	2.92	*	3.6463
**5f**	2.60	2.68	*	3.913
**5g**	2.48	2.63	2.48	3.5674

* Not recorded.

**Table 4:** Electrochemical properties: onset oxidation (E_ox onset_) and reduction (E_red onset_) potentials of NI derivatives relative to the ferrocene/ferrocenium redox couple, electrochemical electron affinity (EA).

Code	E_red onset_	E_1/2_(NI^n^/NI^−1^)	E_ox onset_	EA
	[V]	[V]	[V]	[eV]
**3a**	−1.66	−1.76	*	3.14
**3b**	−1.67	−1.75	*	3.13
**3c**	−1.69	−1.78	*	3.11
**3d**	−1.61	−1.68	*	3.19
**3e**	−1.65	−1.74	*	3.15
**3f**	−1.66	−1.74	*	3.14
**3g**	−1.67	−1.74	0.76	3.13
**3h**	−1.52	−1.77	*	3.28
**3i**	−0.99	−1.07	*	3.82
**5a**	−1.73	−1.82	0.81	3.07
**5b**	−1.74	−1.84	0.73	3.06
**5c**	−1.41	−1.60	*	3.40
**5e**	−1.53	−1.63	*	3.27
**5f**	−1.32	−1.73	*	3.48
**5g**	−1.76	−1.85	0.72	3.04

* Not recorded.

**Table 5:** Performance of test batteries with chosen materials used as a cathode.

	Specific Capacity [mAh/g]		Charge/Discharge Efficiency
	Initial Cycle	After 100 Cycles	[%]
**3i**	47/20	30/14	43–50
**5e**	36/17	26/17	57–66
**5a**	55/17	36/14	33–39

## Data Availability

The data underlying this study are available within the article and Supplementary Material.

## Supplementary Materials

The following supporting information can be downloaded at: https://www.mdpi.com/article/10.3390/molecules31071178/s1.
