# Supplementary material for: N-Hydroxyalkyl and 4-Substituted-N-(hydroxyhexyl)-1,8-naphthalimides: Synthesis and Impact of Molecular Structure on Electrochemical and Photophysical Properties
Source: Molecules. 2026 Apr 2;31(7):1178. doi: 10.3390/molecules31071178 (PMC13074862; doi:10.3390/molecules31071178)
Supplement: Supplementary file 1 [file molecules-31-01178-s001.zip › Suplementary information Manuscript .pdf]

Supplementary Information:

N-Hydroxyalkyl and 4-Substituted-N-(hydroxyhexyl)-1,8-naphthalimides: Synthesis and Impact of Molecular Structure on Electrochemical and Photophysical Properties

Ahmed Chelihi[1,2], Ammara Aslam[3,4], Krzysztof Karoń[3], Wojciech Szczepankiewicz[2], Anna Korytkowska-Wałach[2], Krzysztof Walczak[2*], Przemyslaw Ledwon[3*],

[1]*Laboratory of Catalysis and Synthesis in Organic Chemistry, University of Tlemcen, Algeria*

[2]*Department of Organic Chemistry, Bioorganic Chemistry and Biotechnology, Silesian University of Technology, Krzywoustego 4, 44-100 Gliwice, Poland,*

[3]*Department of Physical Chemistry and Technology of Polymers, Faculty of Chemistry, Silesian University of Technology, 9. M. Strzody St., 44-100 Gliwice, Poland*

[4] *PhD School, Silesian University of Technology, 2a Akademicka str., 44-100 Gliwice, Poland*

**Corresponding Authors: przemyslaw.ledwon@polsl.pl; krzysztof.walczak@polsl.pl*

Content

1. Table S1 of the results of the synthesis of 3a-i compounds 2
2. Table S2 of the results of the synthesis of 5a-i compounds 3
3. ^{1}H NMR, ^{13}C NMR spectra and HRMS spectra 4
4. UV-Vis spectra 20
5. Cyclic Voltammetry 22
6. DFT calculations 25

1. **Table S1 of the results of the synthesis of 3a-i compounds**

naphthalic anhydride	Hydroxyalkylamine (2a-g)	Target compounds (3a-h)	Yields %	Mp ^{0}C
		(3a)	89	108-109
		(3b)	87	102-104
		(3c)	84	92-94
		(3d)	85	153-154
		(3e)	92	132-134
		(3f)	78	192-194
	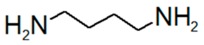	(3g)	87	198-200
		(3h)	85	108-110
		(3i)	87	110-112

2. Table S2 of the results of the synthesis of 5a-i compounds

Entry	Nu-Substitution 4a-g	Products 5a-g	Yield (%)	Mp (^{0}C)
3h	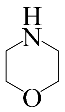	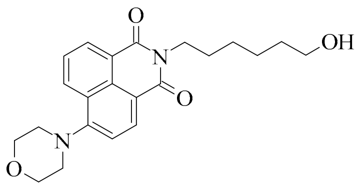 **(5a)**	88	123-124°C
	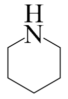	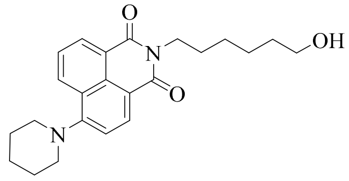 **(5b)**	78	116-118°C
	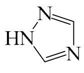	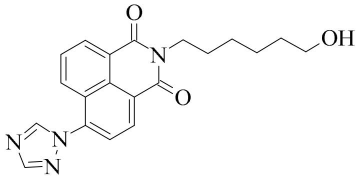 **(5c)**	85	196-198°C
	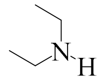	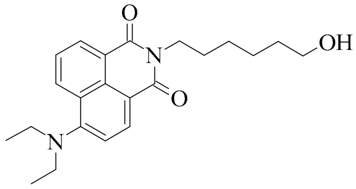 **(5d)**	75	112-113°C
	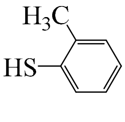	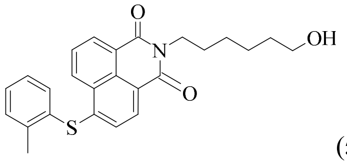 **(5e)**	82	174-175°C
	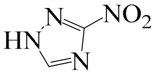	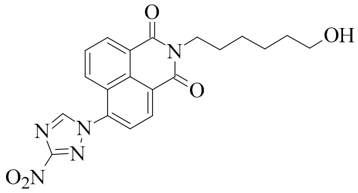 **(5f)**	70	184-185°C
	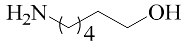	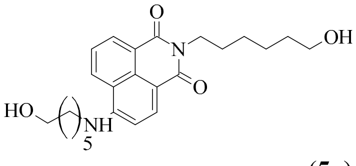 **(5g)**	88	86-88

3. ^{1}H NMR, ^{13}C NMR spectra and HRMS spectra

2-(4-Hydroxybutyl)-1H-benzo[*de*]isoquinoline-1,3(2*H*)-dione (3a)

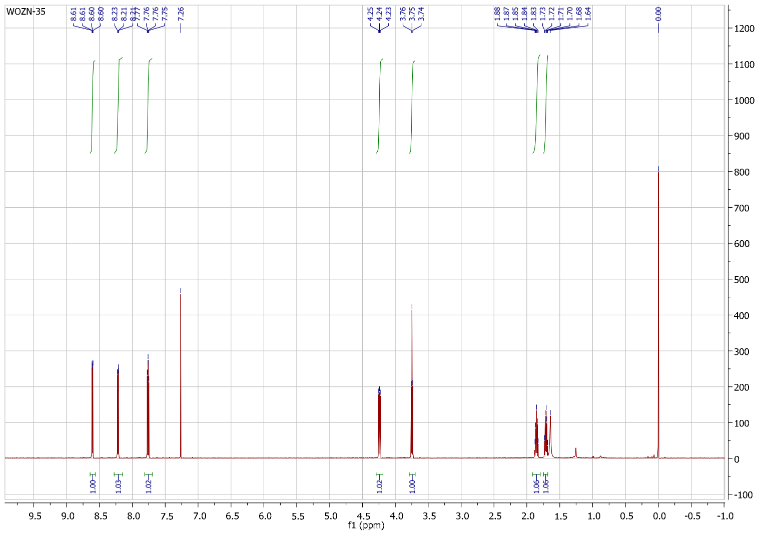

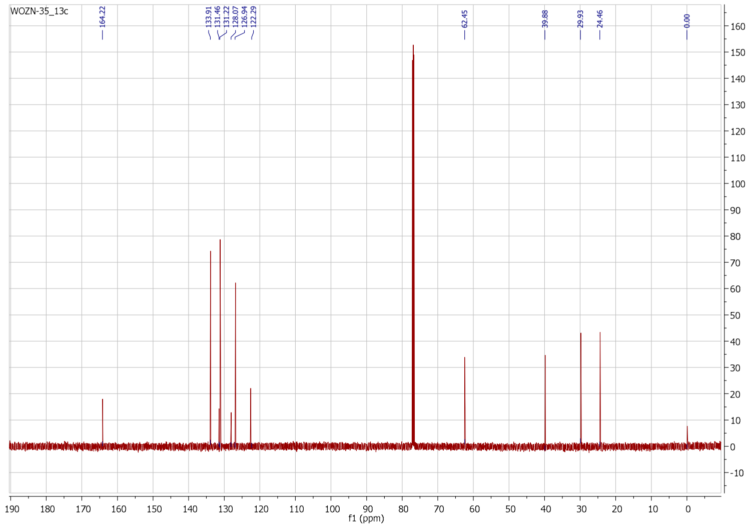

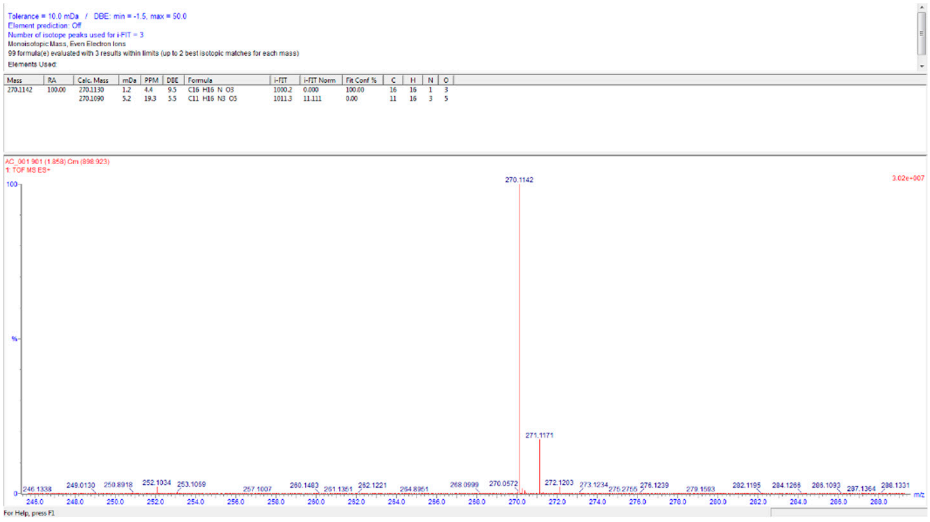

2-(5-Hydroxypentyl)-1*H*-benzo[*de*]isoquinoline-1,3(2*H*)-dione (3b)

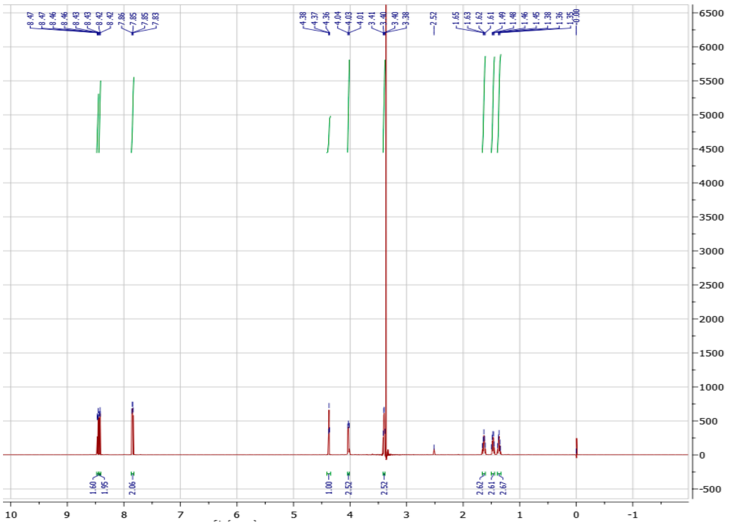

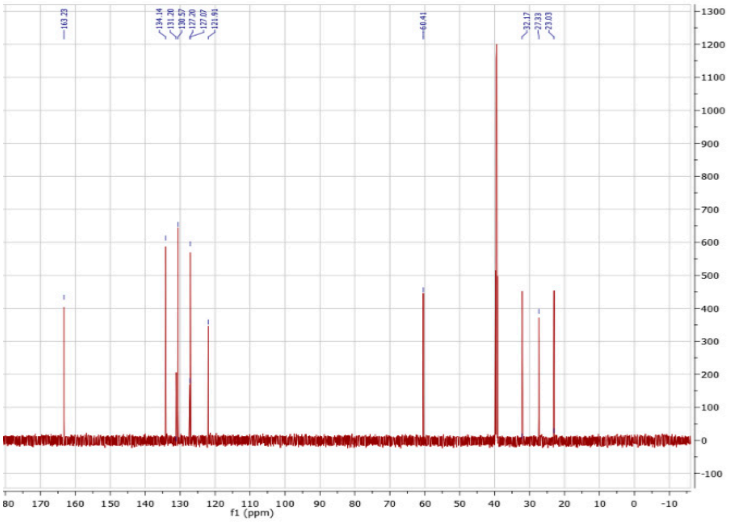

Tolerance = 10.0 mDa / DBE: min = -1.5, max = 50.0
Element prediction: Off
Number of isotope peaks used for i-FIT = 3
Monoisotopic Mass, Even Electron Ions
103 formula(e) evaluated with 3 results within limits (up to 2 best isotopic matches for each mass)
Elements Used:

Mass	RA	Calc. Mass	mDa	PPM	DBE	Formula	i-FIT	i-FIT Norm	Fit Conf %	C	H	N	O
284.1292	100.00	284.1287	0.5	1.8	9.5	C17 H18 N O3	933.6	0.000	99.98	17	18	1	3
		284.1246	4.6	16.2	5.5	C12 H18 N3 O5	942.3	8.628	0.02	12	18	3	5

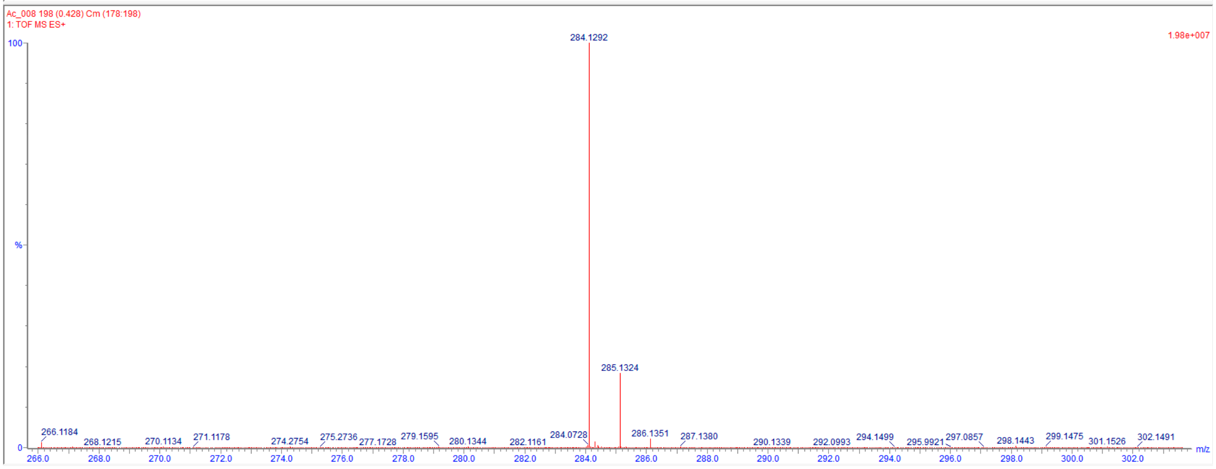

2-(6-Hydroxyhexyl)-1*H*-benzo[*de*]isoquinoline-1,3(2*H*)-dione (3c)

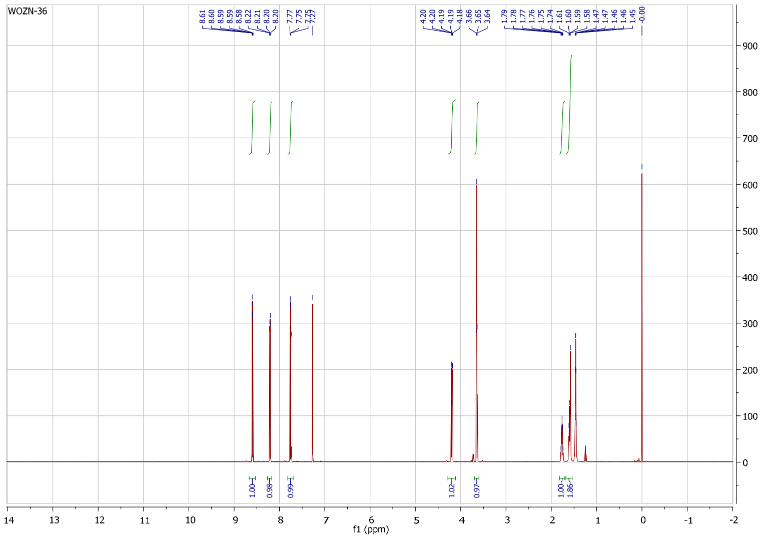

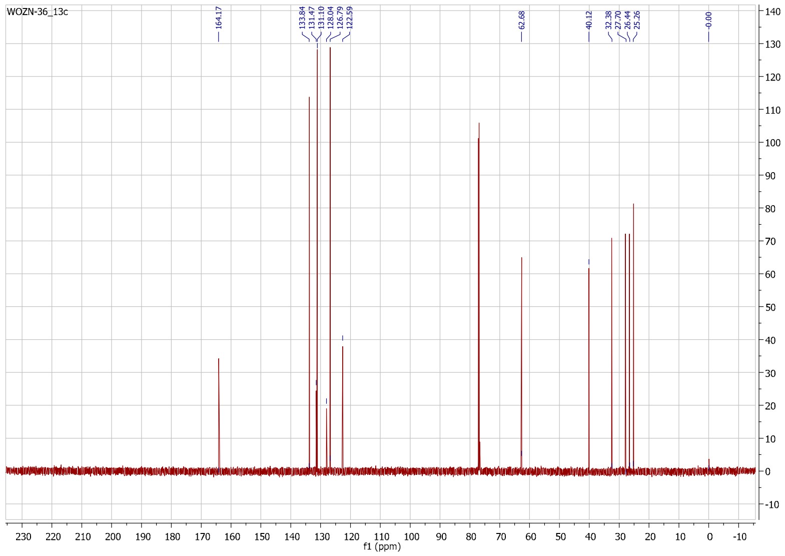

Tolerance = 10.0 mDa / DBE: min = -1.5, max = 50.0
Element prediction: Off
Number of isotope peaks used for i-FIT = 3
Monoisotopic Mass, Even Electron Ions
109 formula(e) evaluated with 3 results within limits (up to 2 best isotopic matches for each mass)
Elements Used:

Mass	RA	Calc. Mass	mDa	PPM	DBE	Formula	i-FIT	i-FIT Norm	Fit Conf %	C	H	N	O
298.1437	100.00	298.1443	-0.6	-2.0	9.5	C18 H20 N O3	890.4	0.004	99.62	18	20	1	3
		298.1403	3.4	11.4	5.5	C13 H20 N3 O5	896.0	5.561	0.38	13	20	3	5

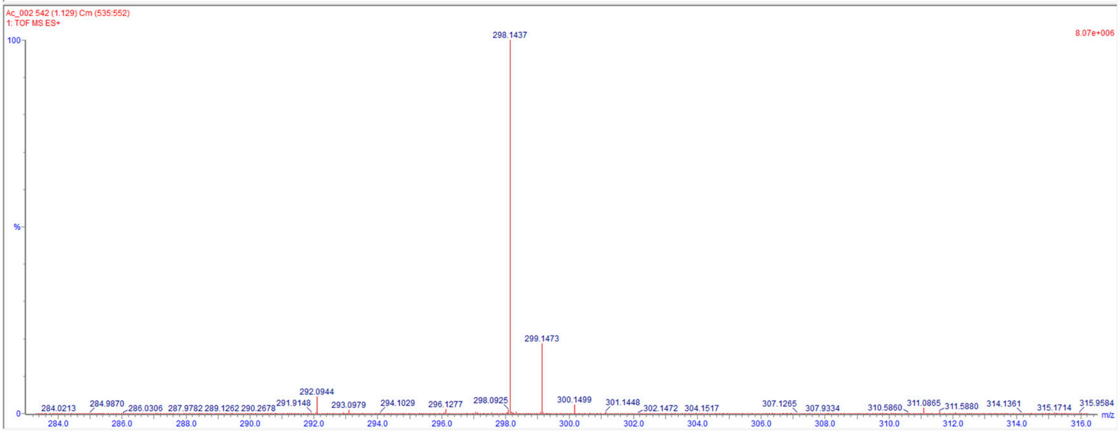

2-(2,3-Dihydroxypropyl)-1*H*-benzo[*de*]isoquinoline-1,3(2*H*)-dione (3d)

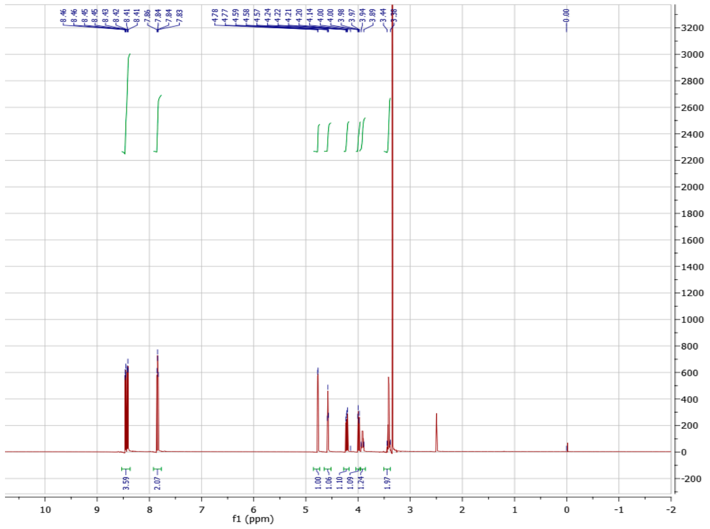

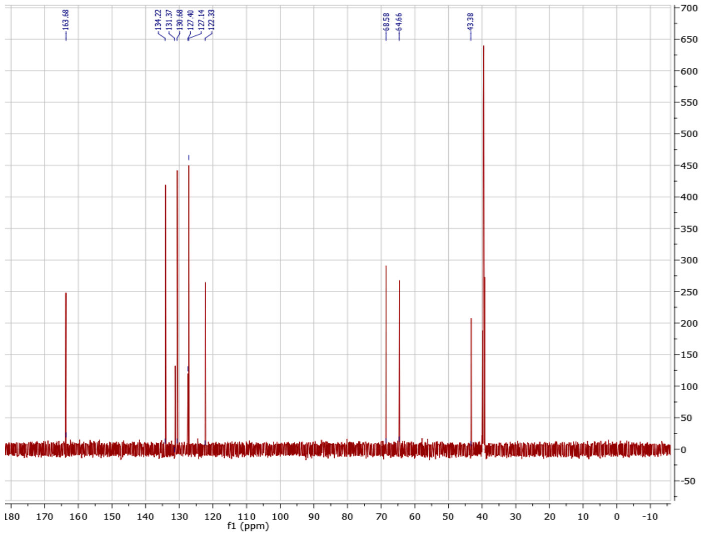

Tolerance = 10.0 mDa / DBE: min = -1.5, max = 50.0
Element prediction: Off
Number of isotope peaks used for i-FIT = 3
Monoisotopic Mass, Even Electron Ions
32 formula(e) evaluated with 2 results within limits (up to 2 best isotopic matches for each mass)
Elements Used:

Mass	RA	Calc. Mass	mDa	PPM	DBE	Formula	i-FIT	i-FIT Norm	Fit Conf %	C	H	N	O
272.0924	100.00	272.0923	0.1	0.4	9.5	C15 H14 N O4	35.3	0.021	97.97	15	14	1	4
		272.0995	-7.1	-26.1	5.5	C9 H14 N5 O5	39.2	3.896	2.03	9	14	5	5

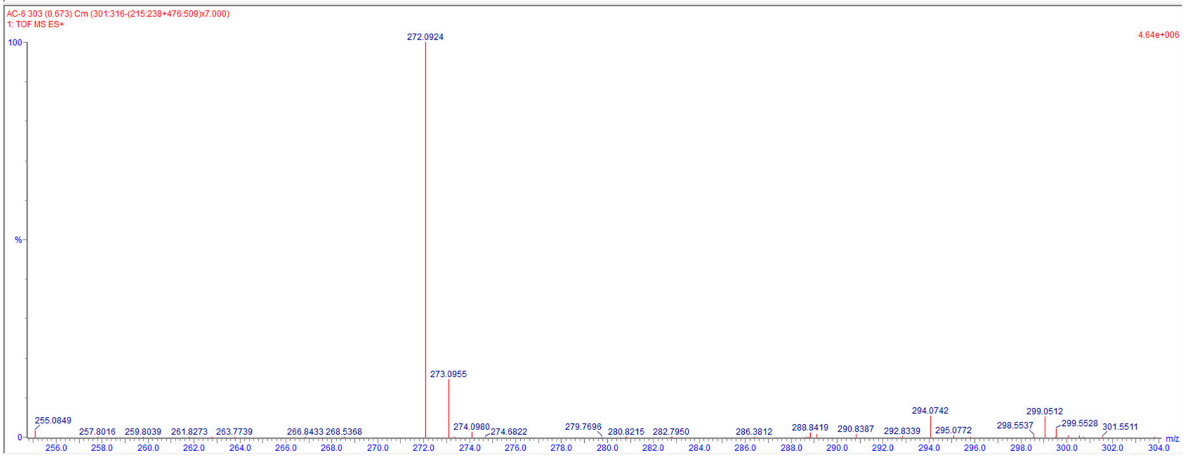

2-(1,3-Dihydroxypropan-2-yl)-1*H*-benzo[*de*]isoquinoline-1,3(2*H*)-dione (3e)

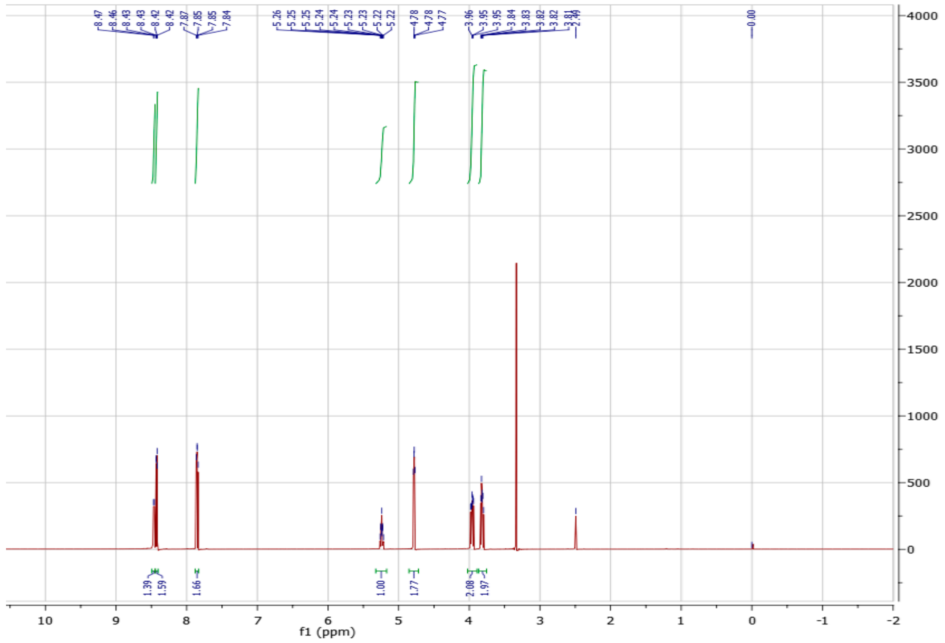

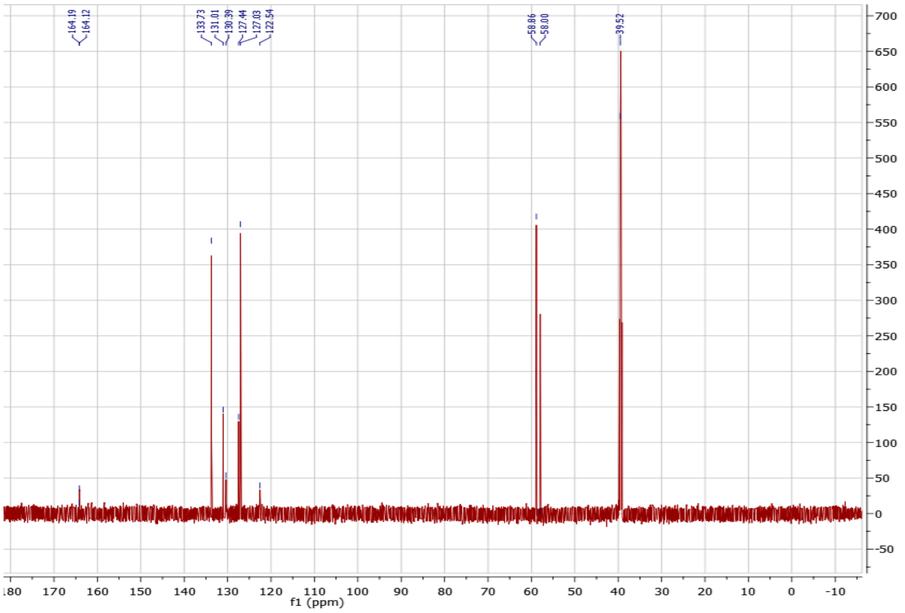

2-(4-(2-hydroxyethyl)phenyl)-1*H*-benzo[*de*]isoquinoline-1,3(2*H*)-dione (3f)

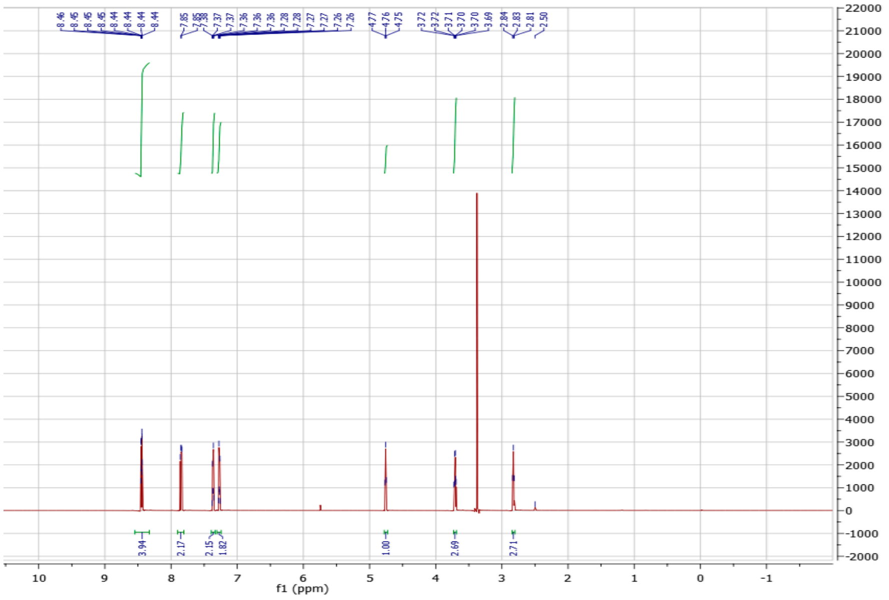

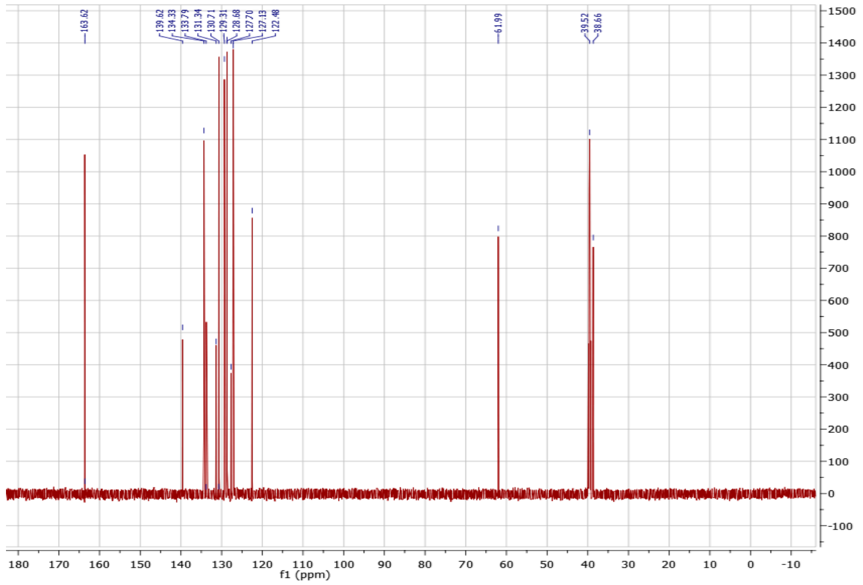

Multiple Mass Analysis: 2 mass(es) processed
Tolerance = 10.0 mDa / DBE: min = -1.5, max = 50.0
Element prediction: Off
Number of isotope peaks used for i-FIT = 3
Monoisotopic Mass, Even Electron Ions
110 formula(e) evaluated with 2 results within limits (up to 2 best isotopic matches for each mass)
Elements Used:

Mass	RA	Calc. Mass	mDa	PPM	DBE	Formula	i-FIT	i-FIT Norm	Fit Conf %	C	H	N	O
318.1141	100.00	318.1130	1.1	3.5	13.5	C20 H16 N O3	890.4	n/a	n/a	20	16	1	3
319.1174	20.43	319.1083	9.1	28.5	13.5	C19 H15 N2 O3	808.3	n/a	n/a	19	15	2	3

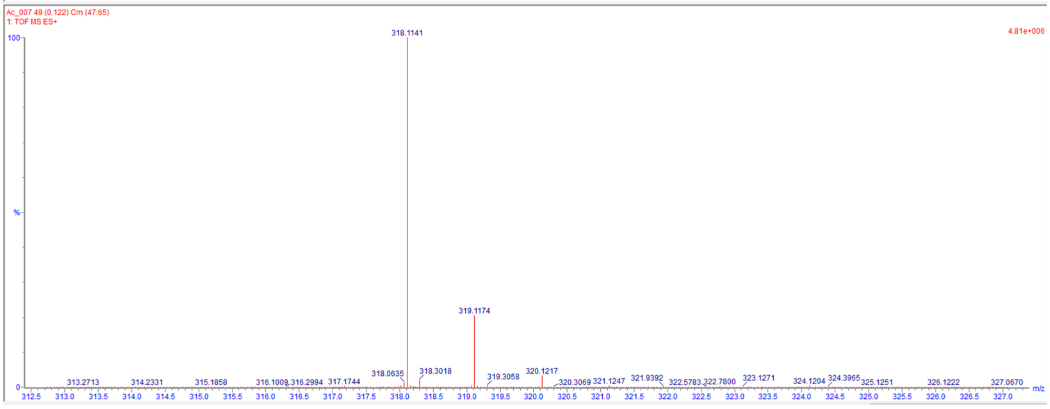

2,2'-(Butane-1,4-diyl)bis(1*H*-benzo[*de*]isoquinoline-1,3(2*H*)-dione) (3g)

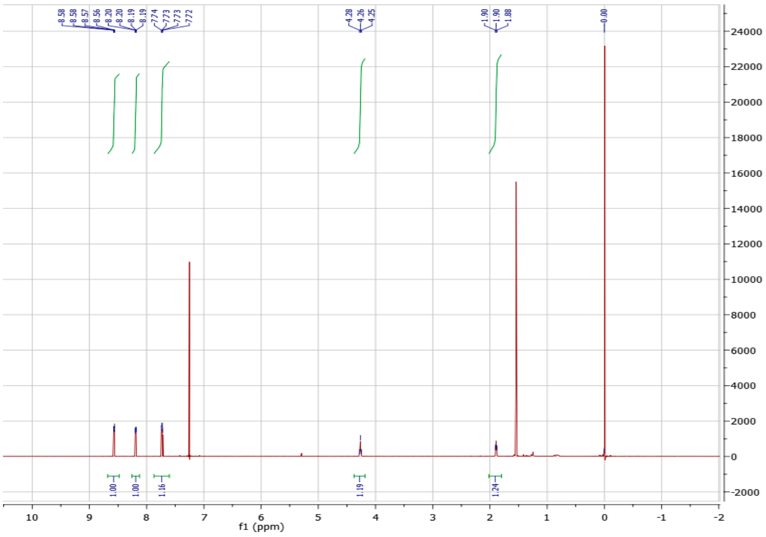

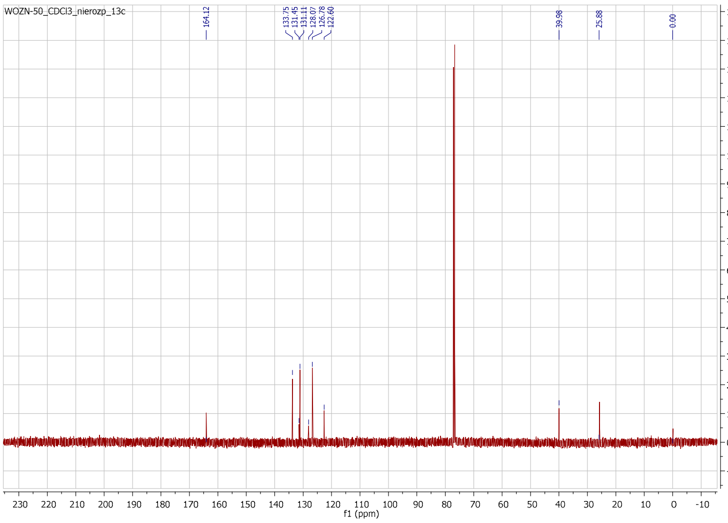

6-Bromo-2-(6-hydroxyhexyl)-1*H*-benzo[*de*]isoquinoline-1,3(2*H*)-dione (3h)

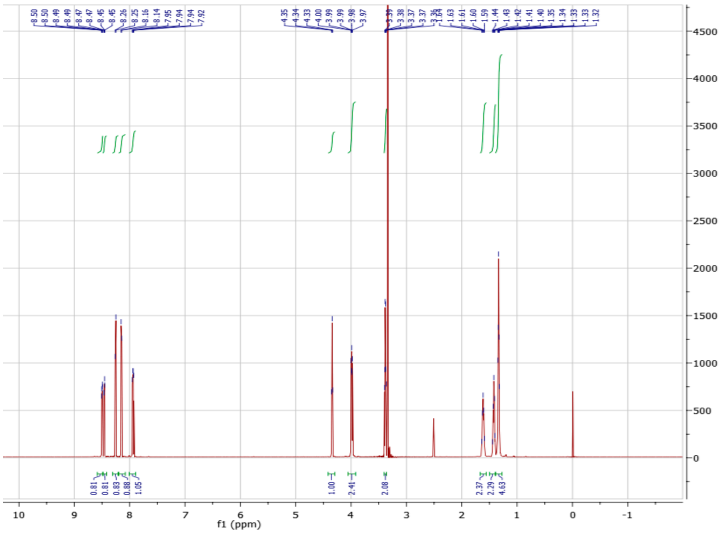

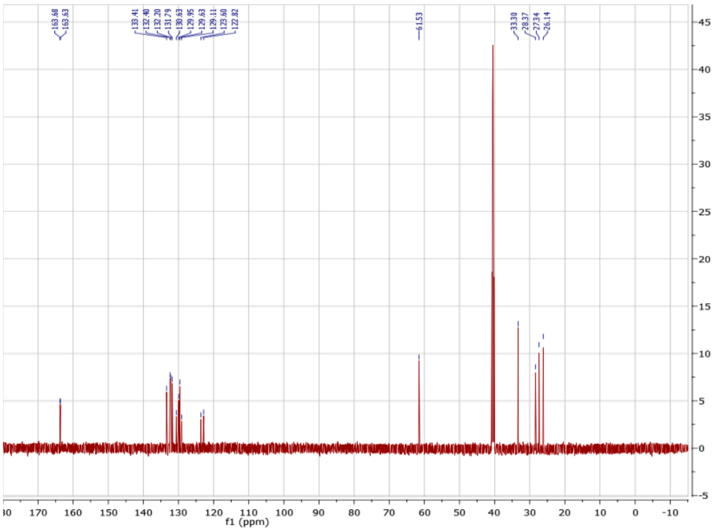

Multiple Mass Analysis: 2 mass(es) processed
Tolerance = 10.0 mDa / DBE: min = -1.5, max = 50.0
Element prediction: Off
Number of isotope peaks used for i-FIT = 3
Monoisotopic Mass, Even Electron Ions
217 formula(e) evaluated with 8 results within limits (up to 2 best isotopic matches for each mass)
Elements Used:

Mass	RA	Calc. Mass	mDa	PPM	DBE	Formula	i-FIT	i-FIT Norm	Fit Conf %	C	H	N	O	Br
376.0528	100.00	376.0548	-2.0	-5.3	9.5	C18 H19 N O3 Br	763.4	0.040	96.06	18	19	1	3	1
		376.0508	2.0	5.3	5.5	C13 H19 N3 O5 Br	766.6	3.235	3.94	13	19	3	5	1
378.0509	97.51	378.0566	-5.7	-15.1	9.5	C15 H17 N5 O2 Br	728.0	0.676	50.85	15	17	5	2	1
		378.0494	1.5	4.0	13.5	C21 H17 N O Br	728.0	0.710	49.15	21	17	1	1	1

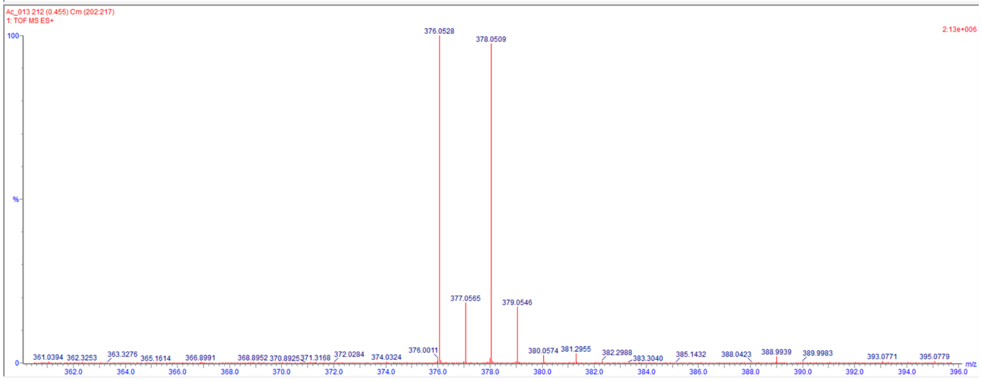

2-(6-Hydroxyhexyl)-6-nitro-1*H*-benzo[*de*]isoquinoline-1,3(2*H*)-dione(3i)

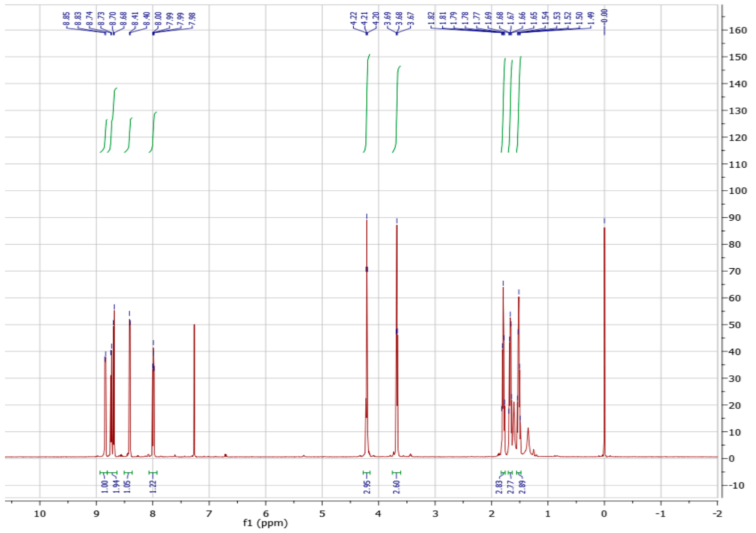

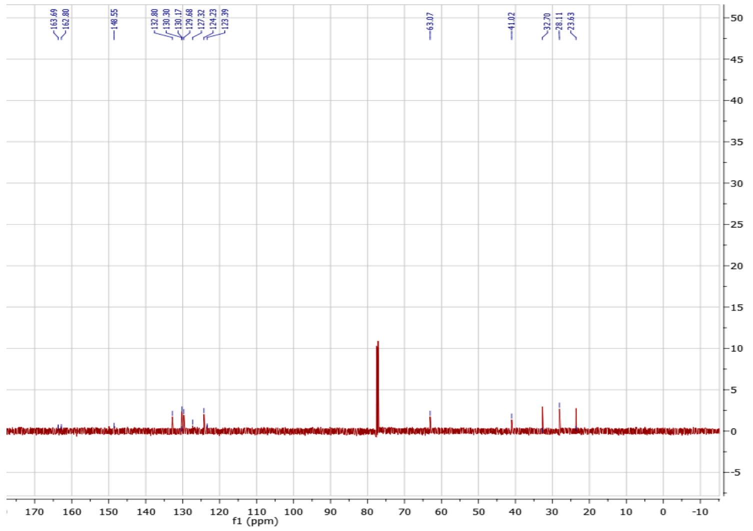

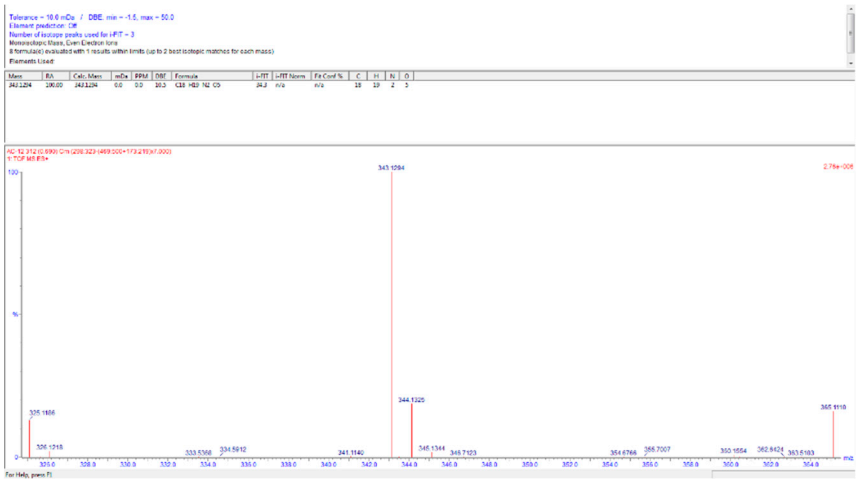

2-(6-Hydroxyhexyl)-6-morpholino-1*H*-benzo[*de*]isoquinoline-1,3(2*H*)-dione (5a)

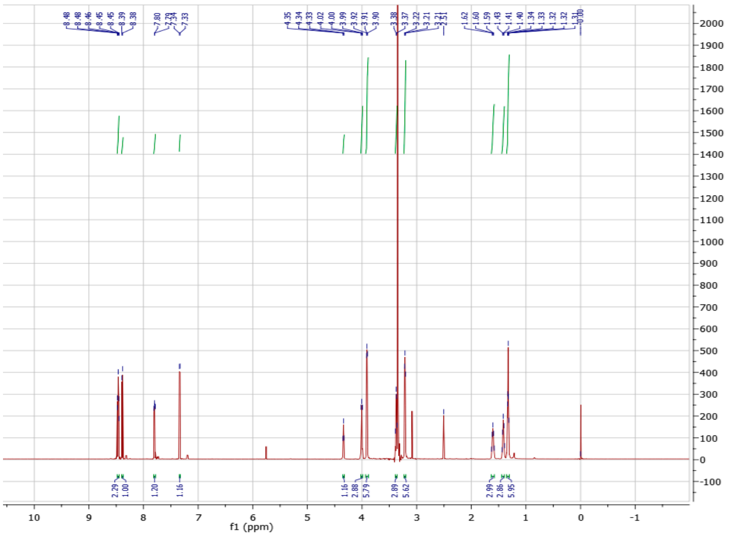

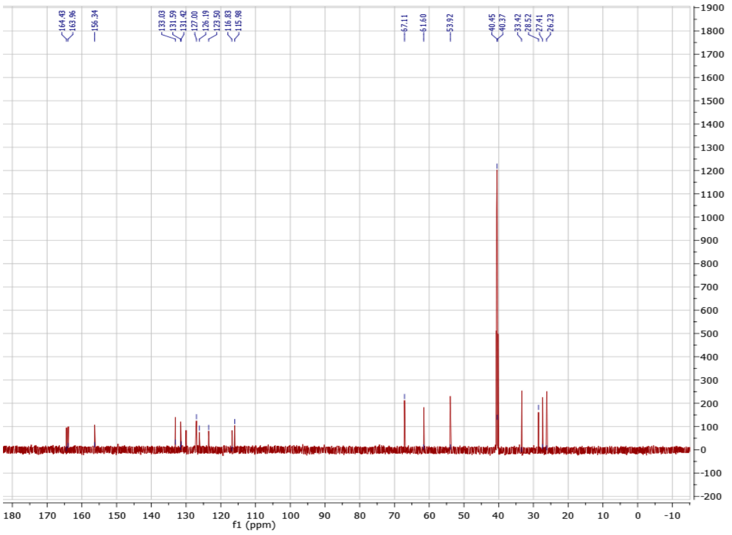

Multiple Mass Analysis: 2 mass(es) processed
Tolerance = 10.0 mDa / DBE: min = -1.5, max = 50.0
Element prediction: Off
Number of isotope peaks used for i-FIT = 3
Monoisotopic Mass, Even Electron Ions
217 formula(e) evaluated with 8 results within limits (up to 2 best isotopic matches for each mass)
Elements Used:

Mass	RA	Calc. Mass	mDa	PPM	DBE	Formula	i-FIT	i-FIT Norm	Fit Conf %	C	H	N	O	Br
376.0528	100.00	376.0548	-2.0	-5.3	9.5	C18 H19 N O3 Br	763.4	0.040	96.06	18	19	1	3	1
		376.0508	2.0	5.3	5.5	C13 H19 N3 O5 Br	766.6	3.235	3.94	13	19	3	5	1
378.0509	97.51	378.0566	-5.7	-15.1	9.5	C15 H17 N5 O2 Br	728.0	0.676	50.85	15	17	5	2	1
		378.0494	1.5	4.0	13.5	C21 H17 N O Br	728.0	0.710	49.15	21	17	1	1	1

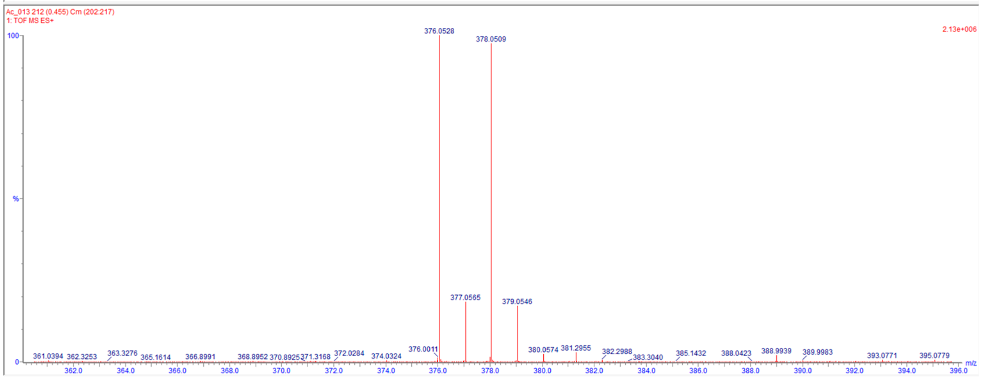

2-(6-Hydroxyhexyl)-6-(piperidin-1-yl)-1*H*-benzo[*de*]isoquinoline-1,3(2*H*)-dione (5b)

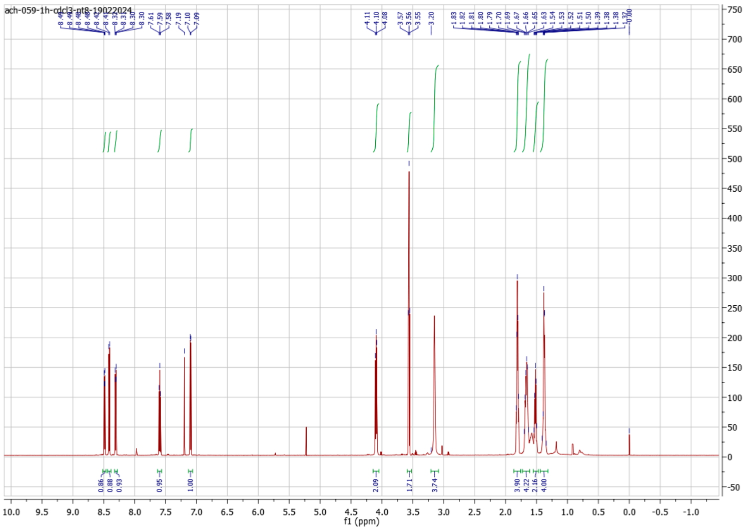

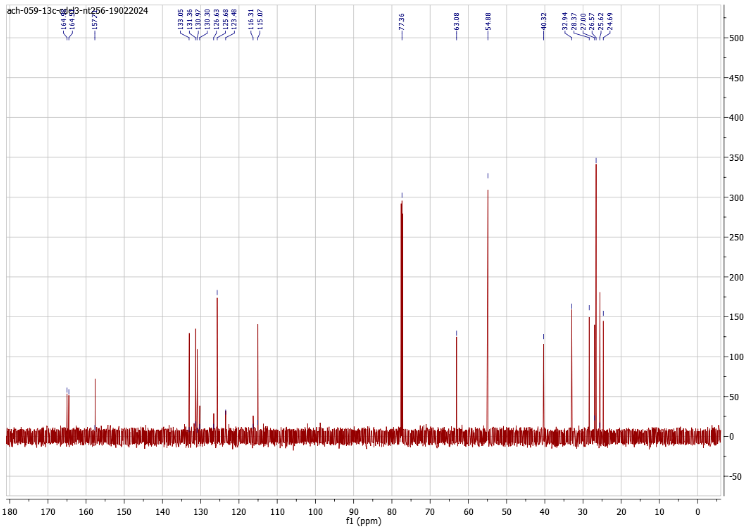

Tolerance = 20.0 PPM / DBE: min = -1.5, max = 50.0
Element prediction: Off
Number of isotope peaks used for i-FIT = 3
Monoisotopic Mass, Even Electron Ions
26 formula(e) evaluated with 2 results within limits (up to 2 best isotopic matches for each mass)
Elements Used:

Mass	RA	Calc. Mass	mDa	PPM	DBE	Formula	i-FIT	i-FIT Norm	Fit Conf %	C	H	N	O	Na
381.2182	100.00	381.2154	2.8	7.3	7.5	C21 H30 N2 O3 Na	982.0	0.003	99.74	21	30	2	3	1
		381.2178	0.4	1.0	10.5	C23 H29 N2 O3	988.0	5.938	0.26	23	29	2	3	

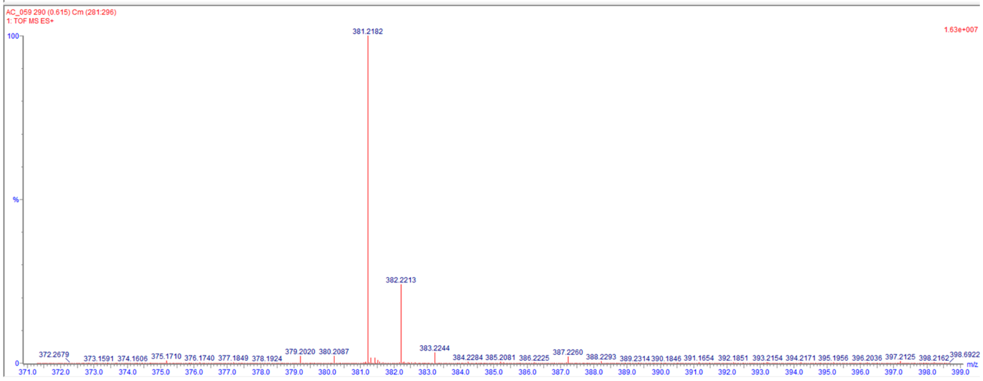

2-(6-Hydroxyhexyl)-6-(1*H*-1,2,4-triazol-1-yl)-1*H*-benzo[*de*]isoquinoline-1,3(2*H*)-dione (5c)

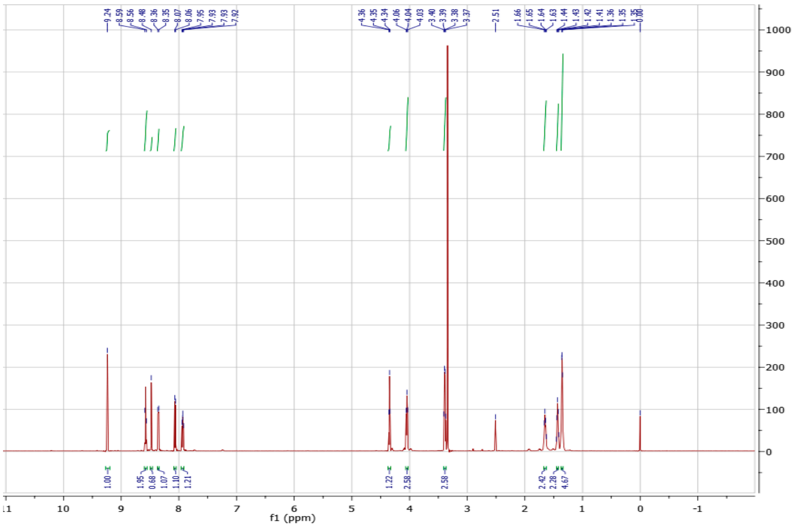

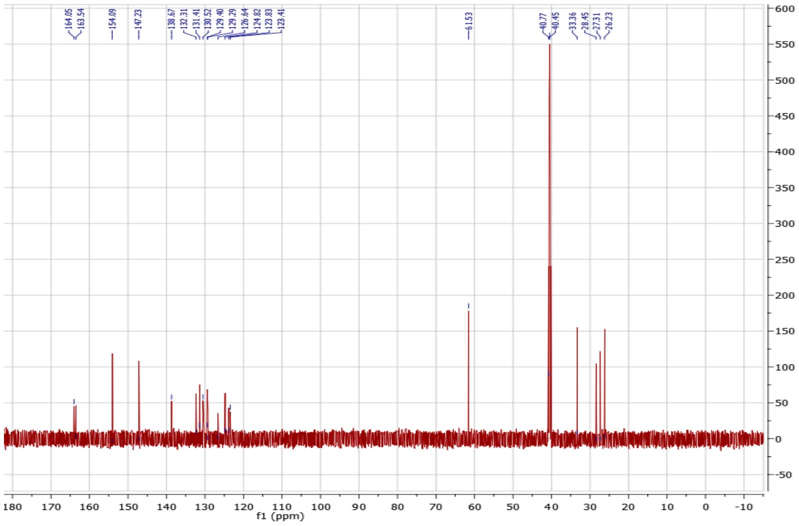

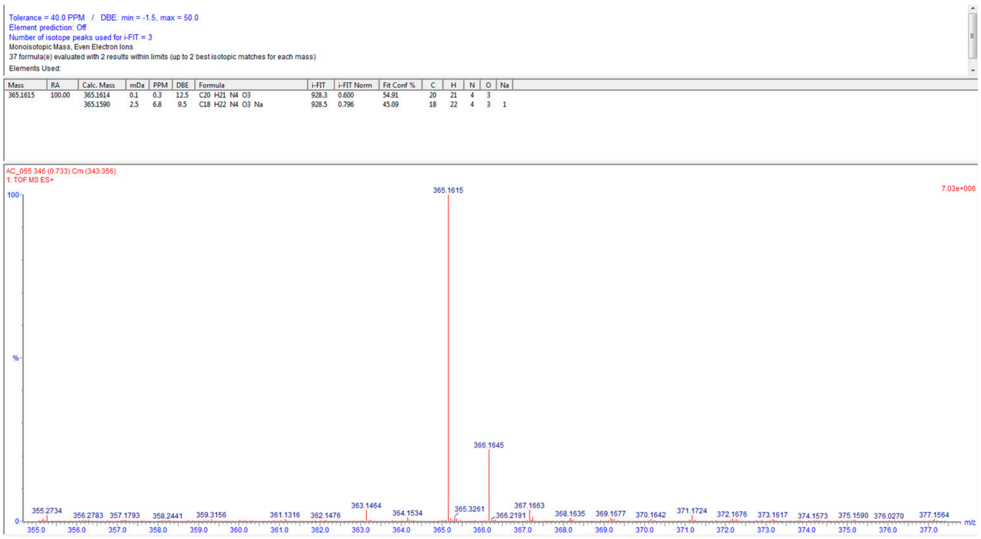

Mass	RA	Calc. Mass	mDa	PPM	DBE	Formula	i-FIT	i-FIT Norm	Fit Conf %	C	H	N	O	Na
365.1615	100.00	365.1614	0.1	0.3	12.5	C20 H21 N4 O3	928.3	0.600	54.91	20	21	4	3	
		365.1590	2.5	6.8	9.5	C18 H22 N4 O3 Na	928.5	0.796	45.09	18	22	4	3	1

6-(Diethylamino)-2-(6-hydroxyhexyl)-1*H*-benzo[*de*]isoquinoline-1,3(2*H*)-dione (5d)

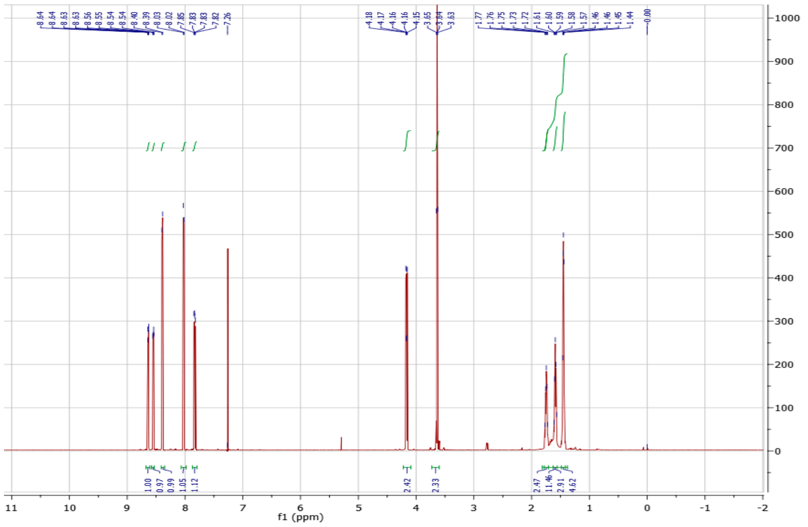

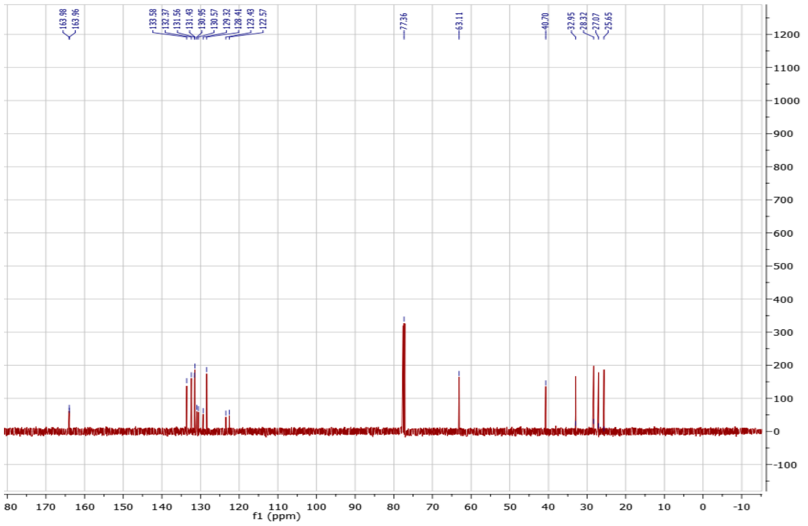

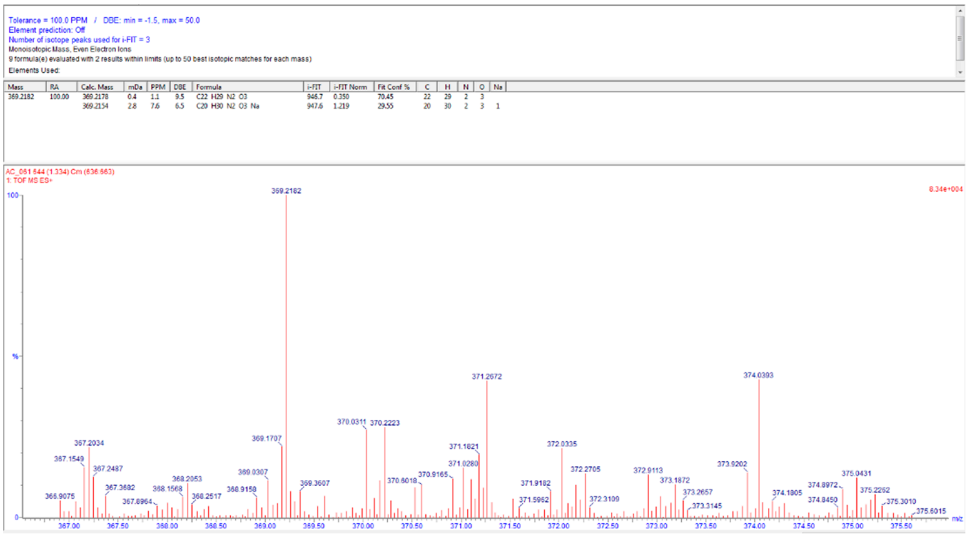

2-(6-Hydroxyhexyl)-6-(*o*-tolylthio)-1*H*-benzo[*de*]isoquinoline-1,3(2*H*)-dione (5e)

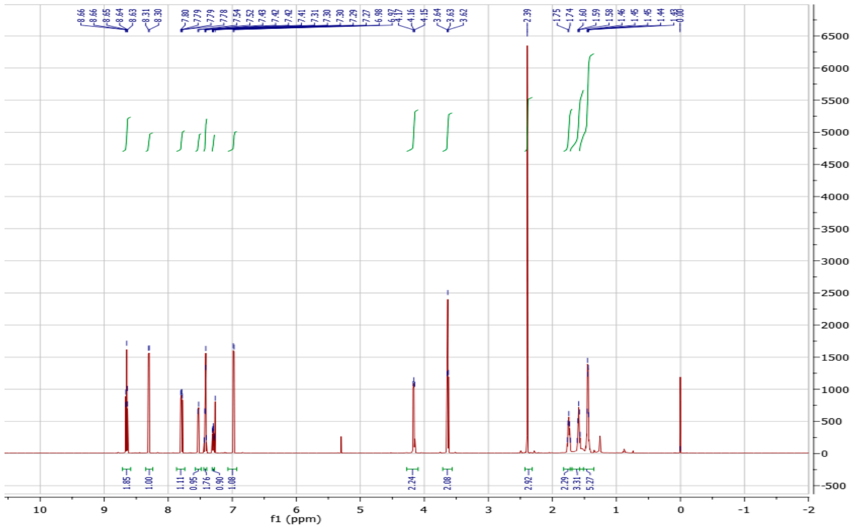

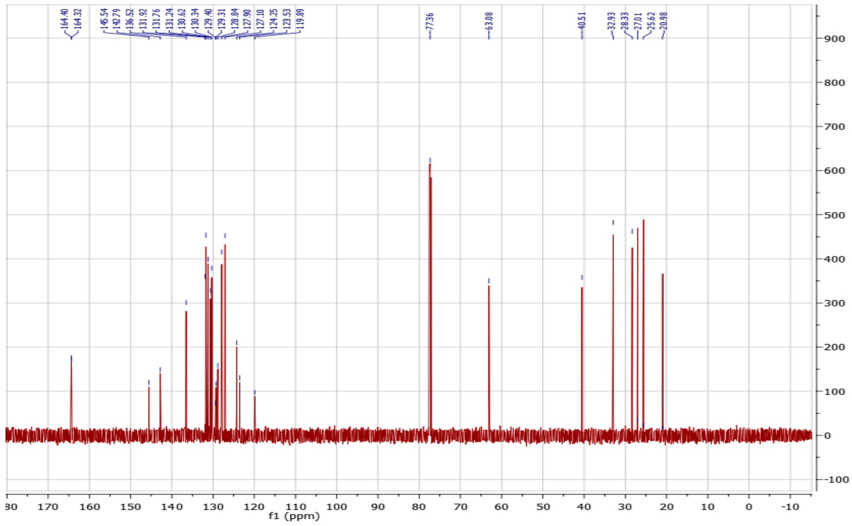

Multiple Mass Analysis: 2 mass(es) processed
Tolerance = 10.0 mDa / DBE: min = -1.5, max = 50.0
Element prediction: Off
Number of isotope peaks used for i-FIT = 3
Monoisotopic Mass, Even Electron Ions
280 formula(e) evaluated with 5 results within limits (up to 2 best isotopic matches for each mass)
Elements Used:

Mass	RA	Calc. Mass	mDa	PPM	DBE	Formula	i-FIT	i-FIT Norm	Fit Conf %	C	H	N	O	S
420.1622	100.00	420.1633	-1.1	-2.6	13.5	C25 H26 N O3 S	768.5	0.008	99.20	25	26	1	3	1
		420.1593	2.9	6.9	9.5	C20 H26 N3 O5 S	773.3	4.833	0.80	20	26	3	5	1
421.1654	27.49	421.1698	-4.4	-10.4	13.5	C23 H25 N4 O2 S	738.2	0.576	56.19	23	25	4	2	1
		421.1586	6.8	16.1	13.5	C24 H25 N2 O3 S	738.5	0.825	43.81	24	25	2	3	1

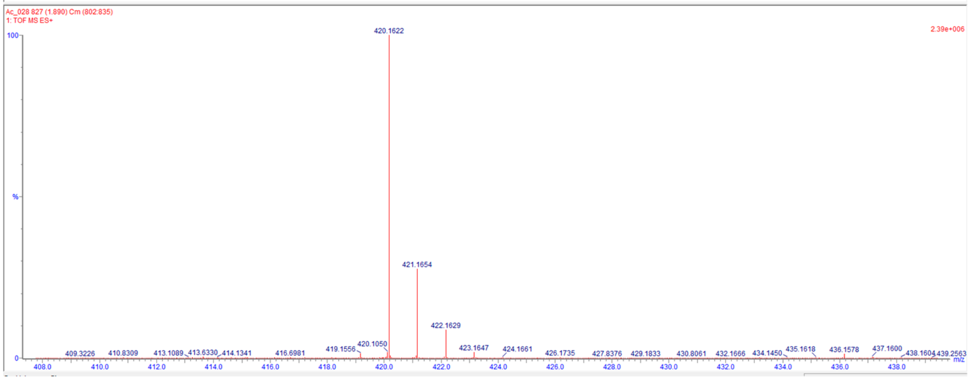

2-(6-Hydroxyhexyl)-6-(3-nitro-1*H*-1,2,4-triazol-1-yl)-1*H*-benzo[*de*]isoquinoline-1,3(2*H*)-dione (5f)

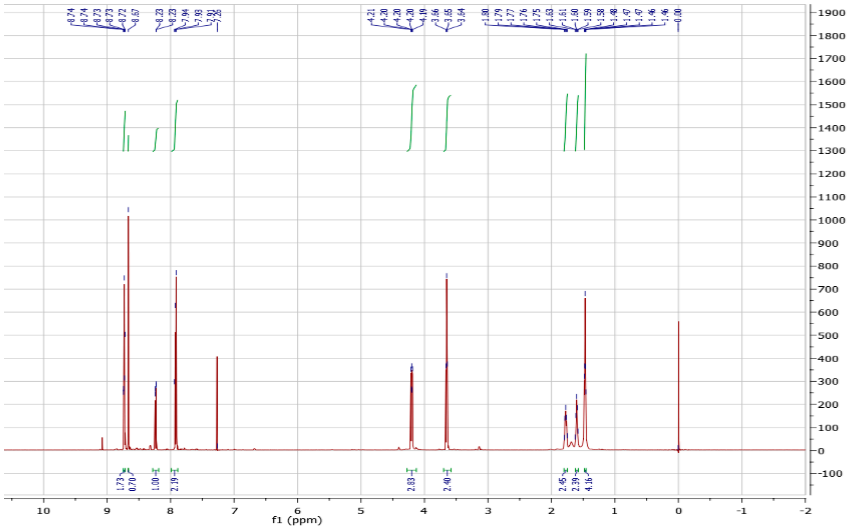

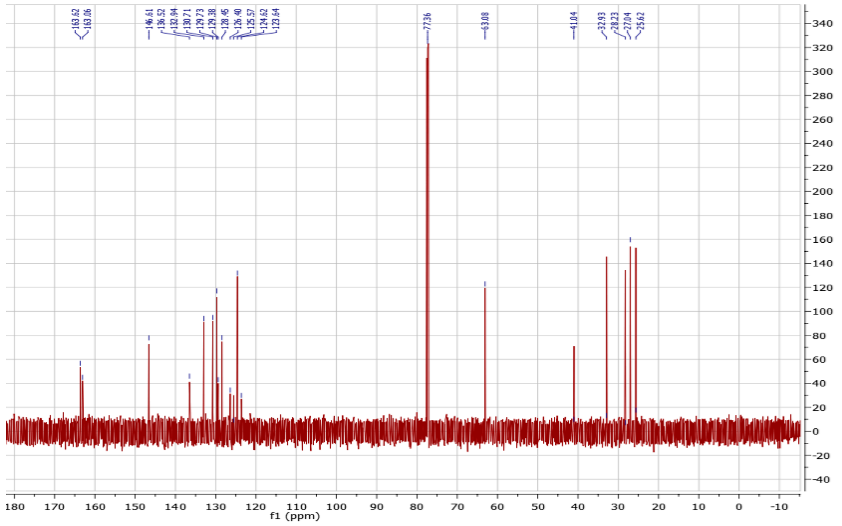

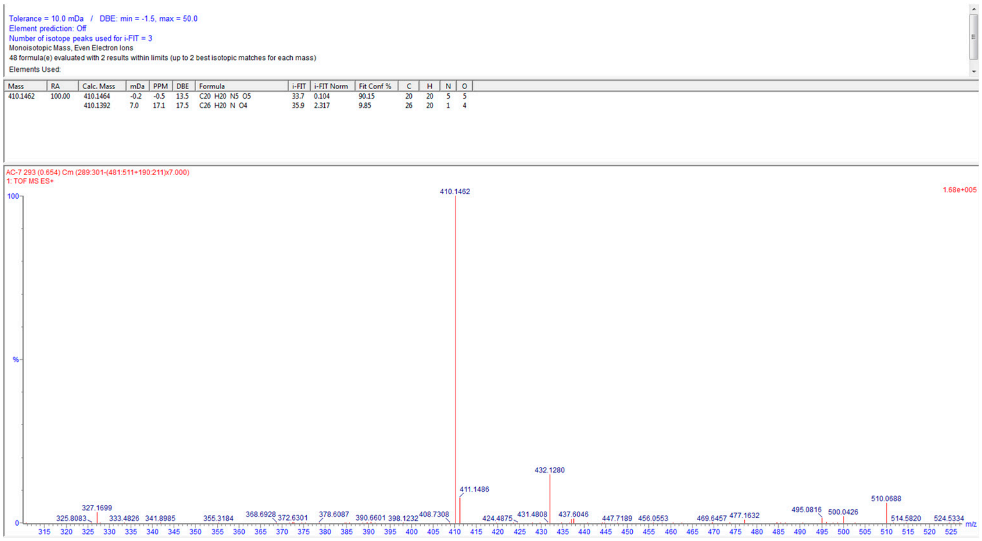

2-(6-Hydroxyhexyl)-6-(6-hydroxyhexylamino)-1*H*-benzo[*de*]isoquinoline-1,3(2*H*)-dione (5g)

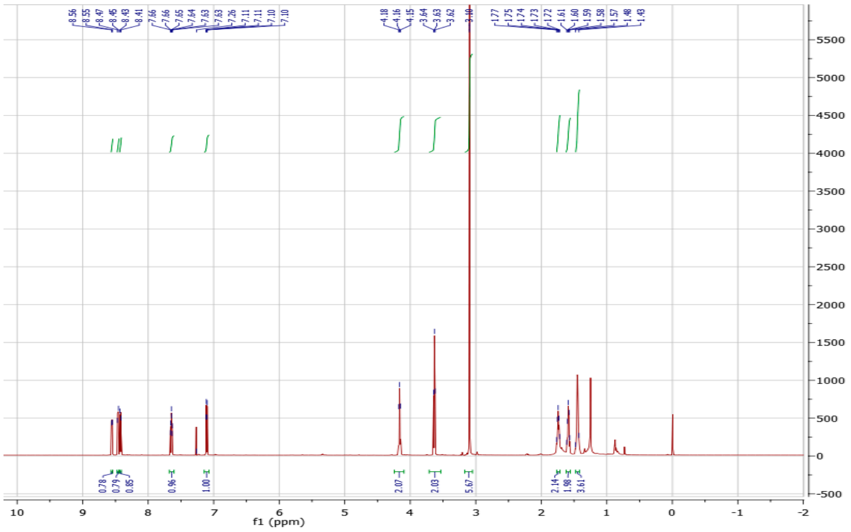

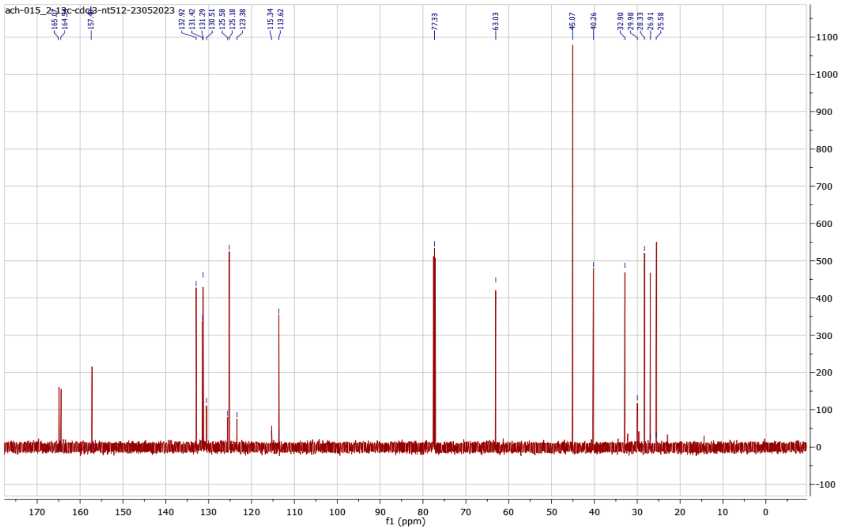
ach-015_2-13c-cdcl3-nt512-23052023
f1 (ppm)

4. UV-Vis spectra

Figure S1 UV-Vis-NIR spectra recorded in ACN, with concentrations ranging from 0.1 mM/ml to 0.5 mM/ml.

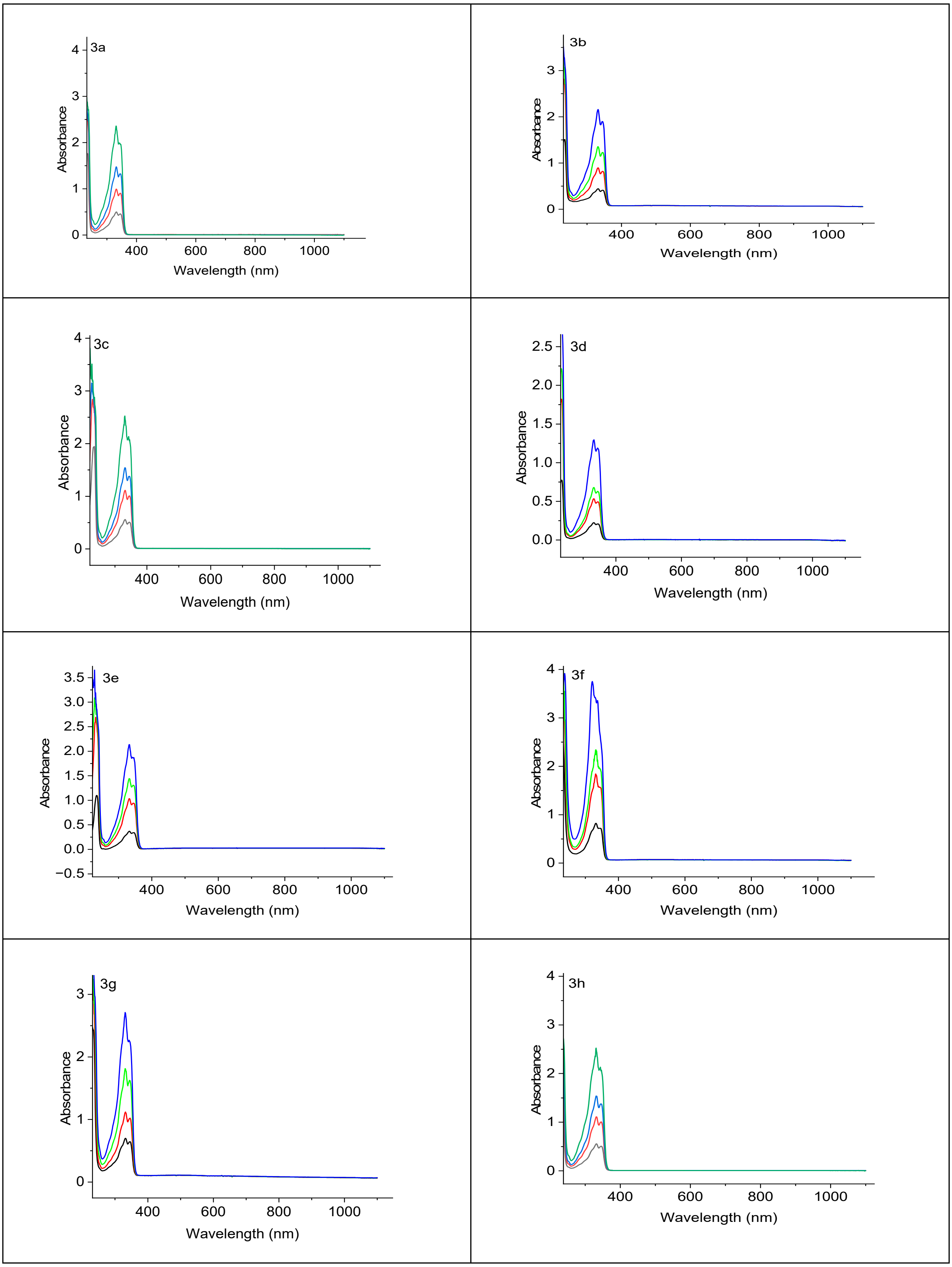

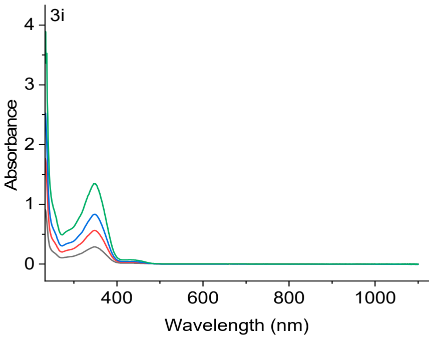
3i
Absorbance
0 1 2 3 4
400 600 800 1000
Wavelength (nm)

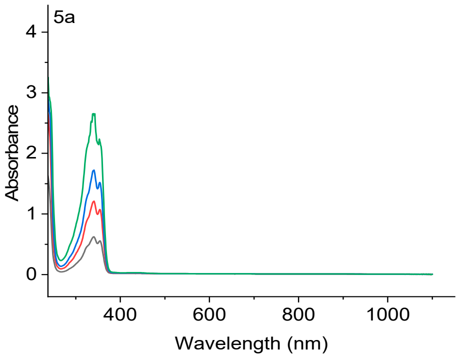
5a
Absorbance
0 1 2 3 4
400 600 800 1000
Wavelength (nm)

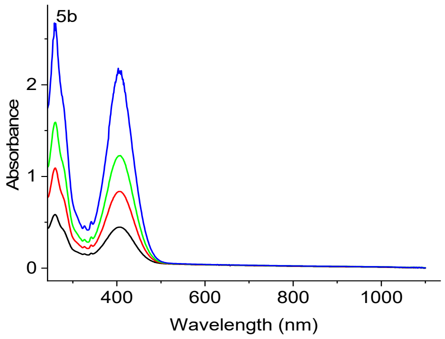
5b
Absorbance
0 1 2
400 600 800 1000
Wavelength (nm)

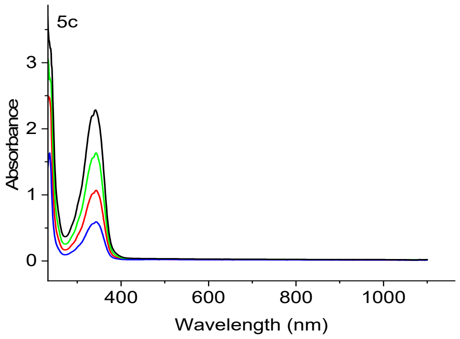
5c
Absorbance
0 1 2 3
400 600 800 1000
Wavelength (nm)

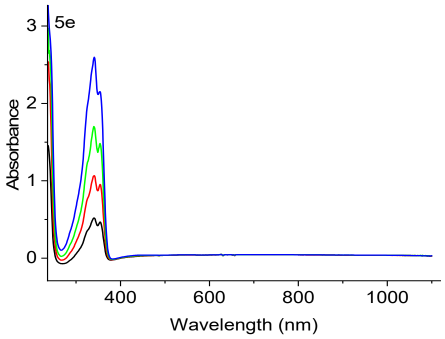
5e
Absorbance
0 1 2 3
400 600 800 1000
Wavelength (nm)

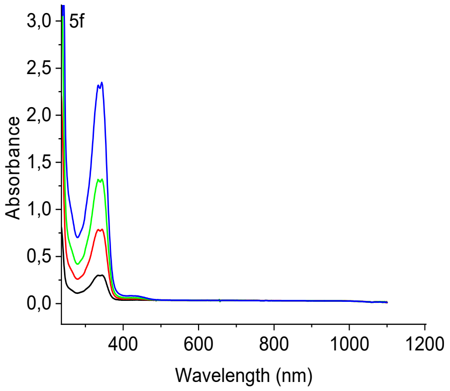
5f
Absorbance
0,0 0,5 1,0 1,5 2,0 2,5 3,0
400 600 800 1000 1200
Wavelength (nm)

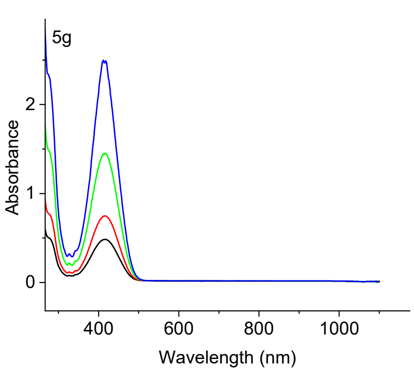
5g
Absorbance
0 1 2
400 600 800 1000
Wavelength (nm)

5. Cyclic Voltammetry

Figure S2 Cyclic voltammetry of all compounds in ACN/Bu_4NPF_6 electrolyte.

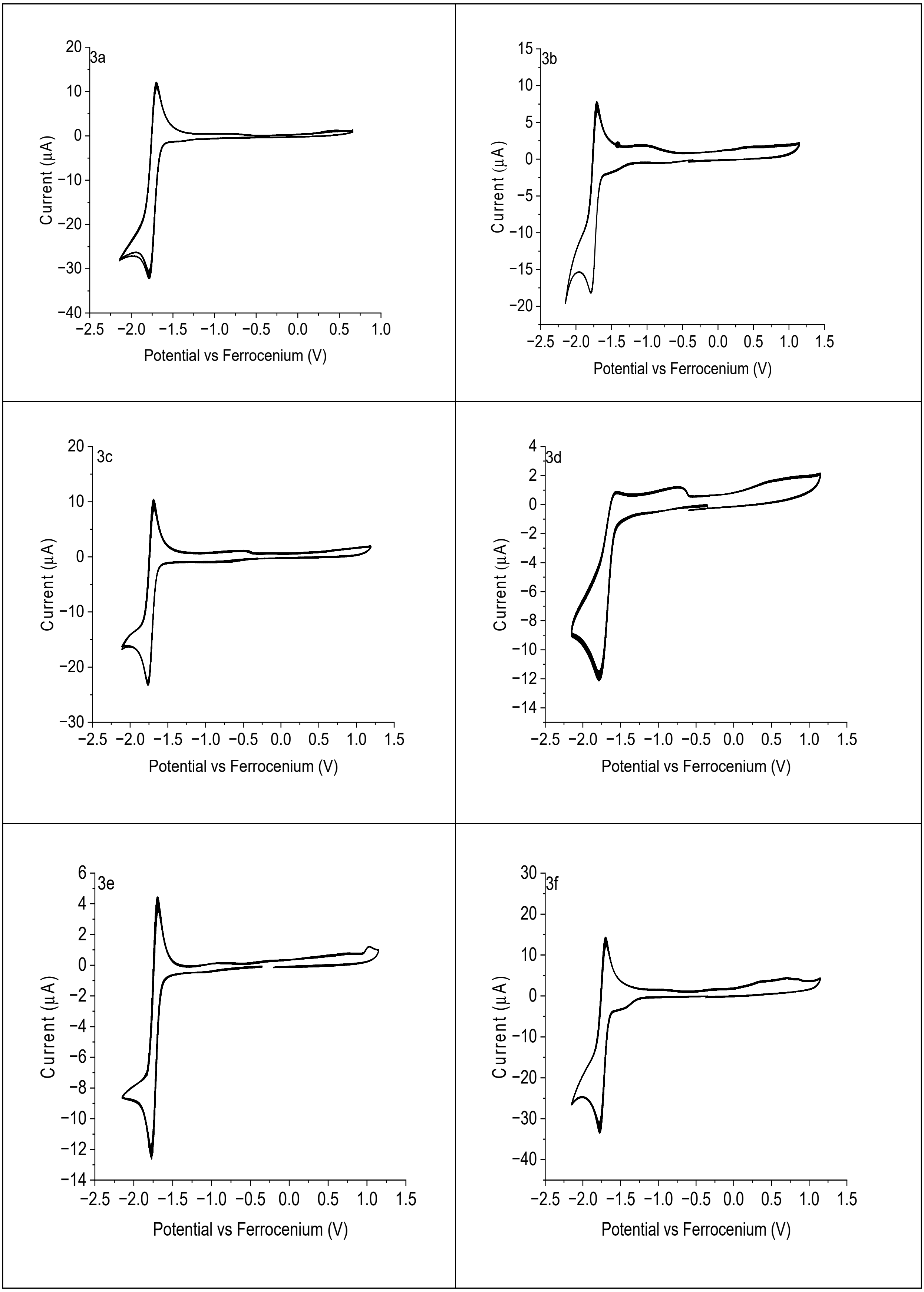

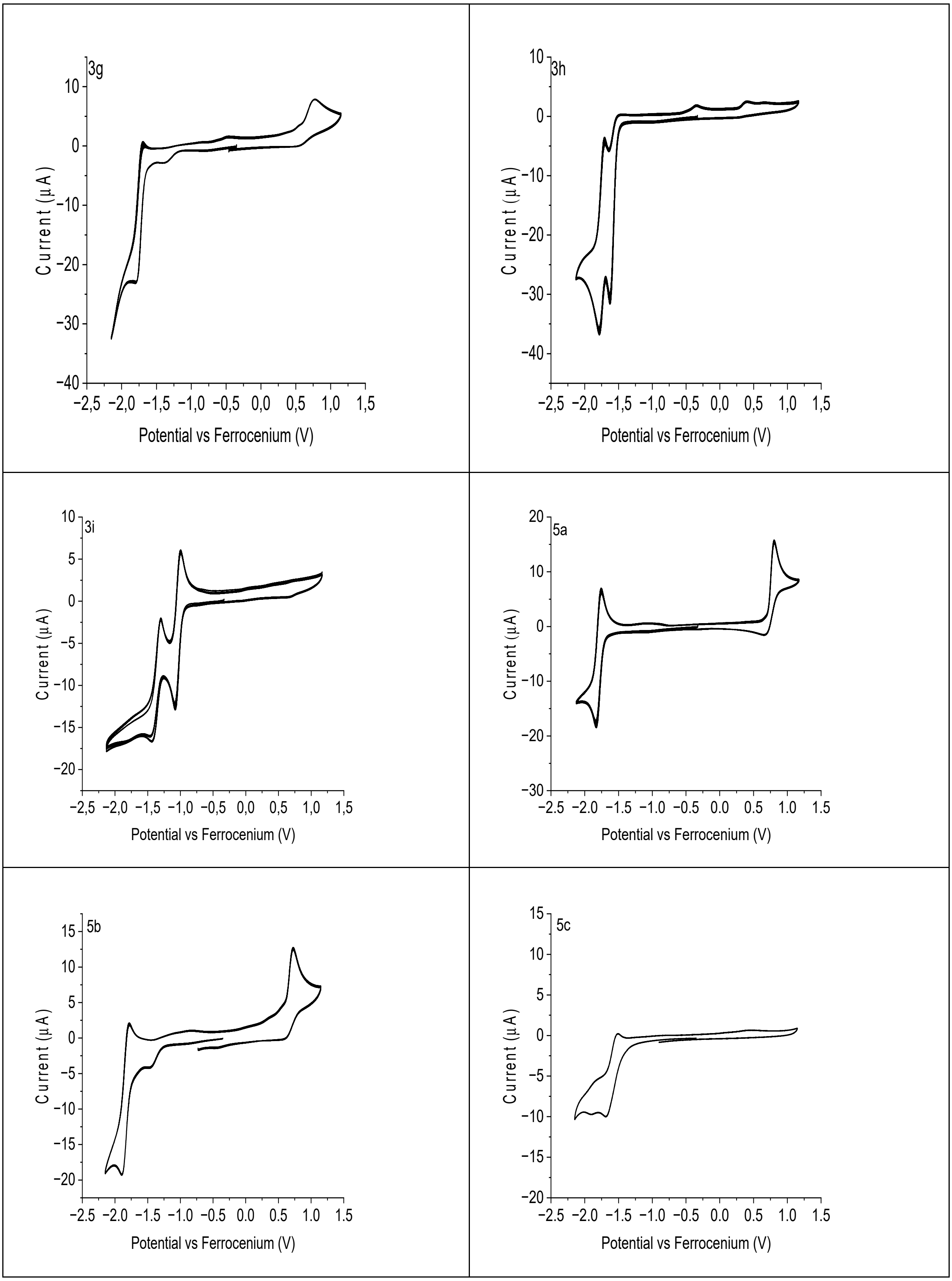
3g
3h
3i
5a
5b
5c
Current (μA)
Potential vs Ferrocenium (V)

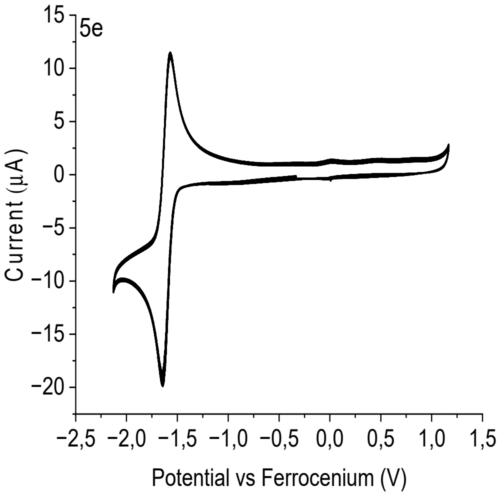
5e
Current (µA)
15
10
5
0
−5
−10
−15
−20
−2,5 −2,0 −1,5 −1,0 −0,5 0,0 0,5 1,0 1,5
Potential vs Ferrocenium (V)

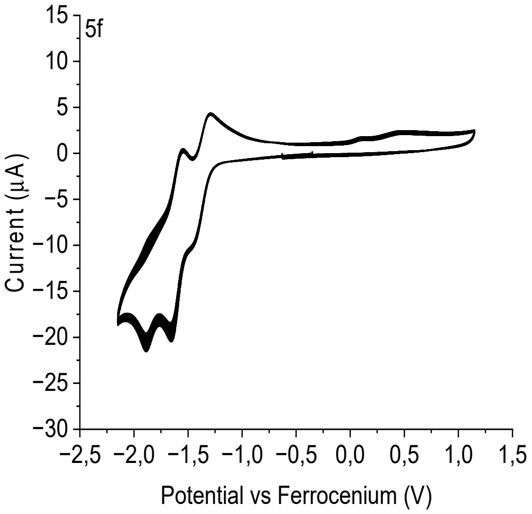
5f
Current (µA)
15
10
5
0
−5
−10
−15
−20
−25
−30
−2,5 −2,0 −1,5 −1,0 −0,5 0,0 0,5 1,0 1,5
Potential vs Ferrocenium (V)

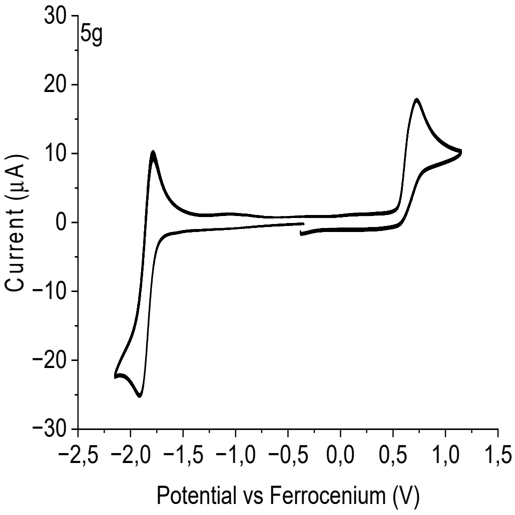
5g
Current (µA)
30
20
10
0
−10
−20
−30
−2,5 −2,0 −1,5 −1,0 −0,5 0,0 0,5 1,0 1,5
Potential vs Ferrocenium (V)

6. DFT calculations

Table S3 DFT-calculated energy levels

code	HOMO-2 (eV)	HOMO-1 (eV)	HOMO (eV)	LUMO (eV)	LUMO+1 (eV)	LUMO+2 (eV)	Eg_{DFT} (eV)
**3c**	-7,0205	-6,8926	-6,4056	-2,3402	-0,6939	-0,5959	4,0654
**3h**	-7,4532	-7,1865	-6,7212	-2,7892	-1,2381	-1,0395	3,932
**3i**	-7,4532	-7,026	-6,9525	-3,2409	-2,0218	-1,2327	3,7116
**5a**	-6,818	-6,682	-5,573	-2,122	-0,614	-0,409	3,451
**5b**	-6,802	-6,739	-5,706	-2,101	-0,53	-0,373	3,605
**5c**	-7,2192	-6,9607	-6,5552	-2,6449	-1,0177	-0,9932	3,9103
**5e**	-6,8981	-6,8518	-5,9049	-2,2585	-0,8626	-0,6776	3,6463
**5f**	-7,4722	-7,0477	-6,9307	-3,0177	-2,4708	-1,3415	3,913
**5g**	-6,7267	-6,6722	-5,5103	-1,9429	-0,468	-0,2014	3,5674

Table S4 3D representation of molecular frontier orbitals of ***3c*, *3h*** *and* ***3i***

	3c	*3h*	*3i*
LUMO+2	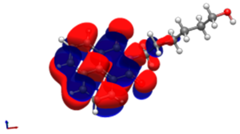	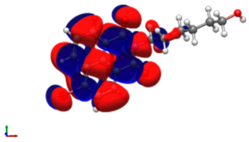	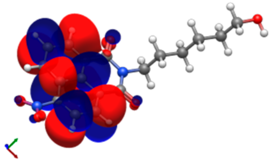
LUMO+1	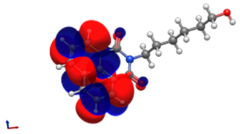	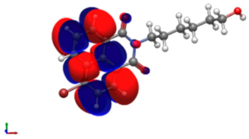	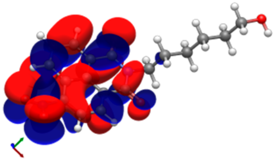
LUMO	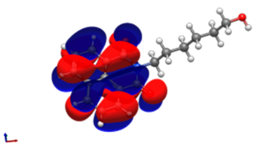	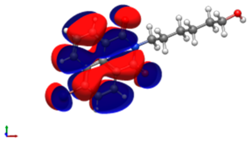	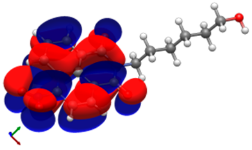
HOMO	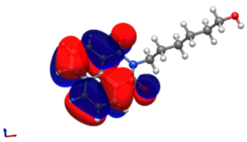	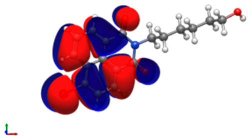	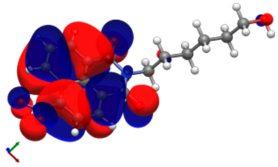
HOMO-1	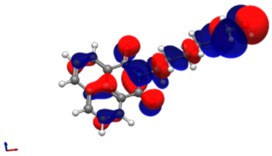	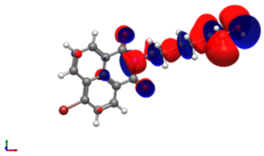	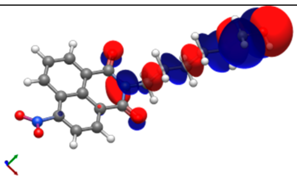
HOMO-2	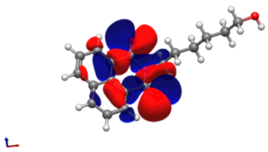	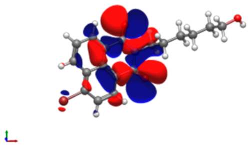	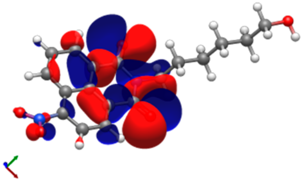

Table S5 3D representation of molecular frontier orbitals of ***5a*****,** ***5b*** *and* ***5c***

	5a	*5b*	***5c***
LUMO+2	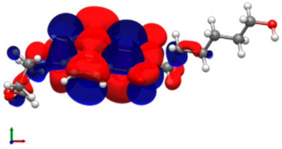	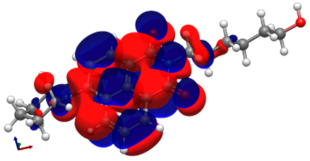	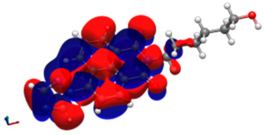
LUMO+1	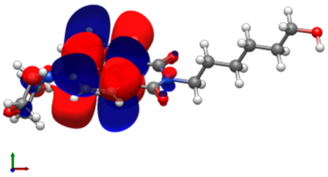	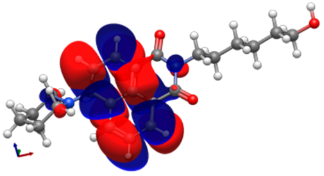	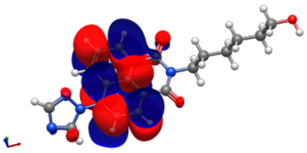
LUMO	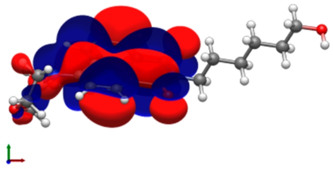	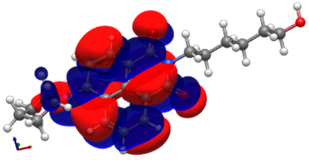	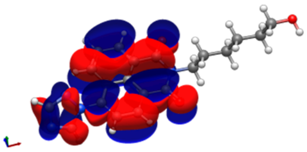
HOMO	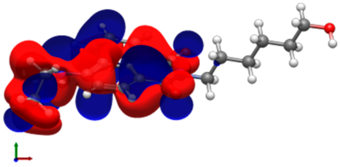	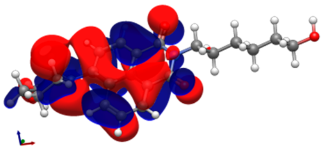	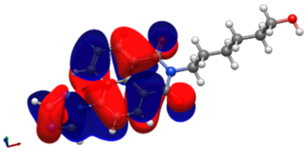
HOMO-1	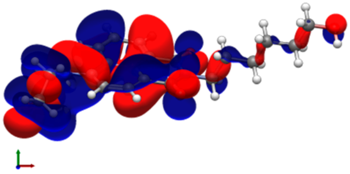	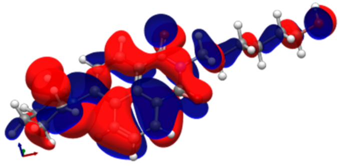	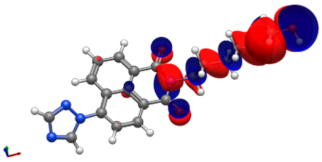
HOMO-2	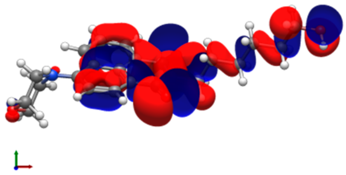	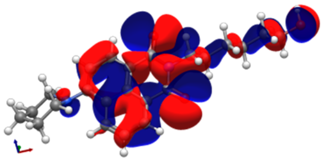	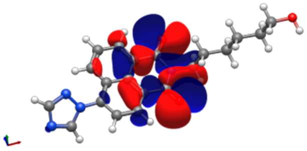

*Table S6 3D representation of molecular frontier orbitals of **5e**, **5f** and **5g** molecules*

	5e	*5f*	*5g*
LUMO+2	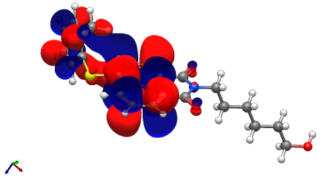	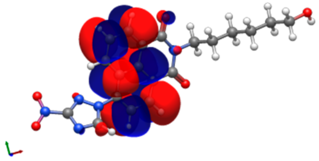	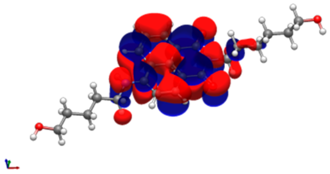
LUMO+1	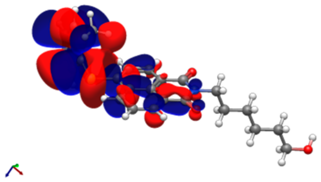	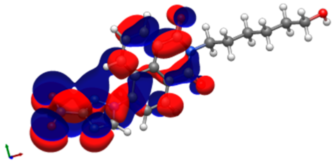	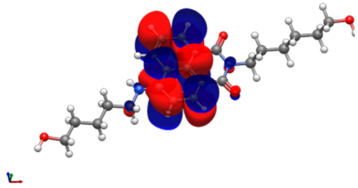
LUMO	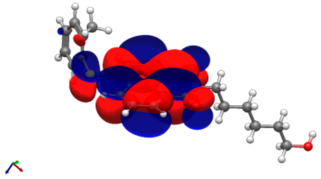	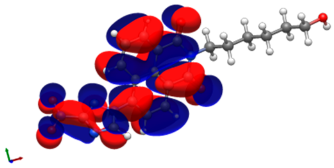	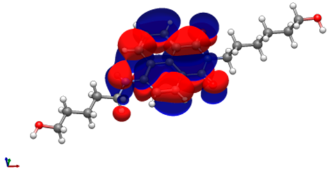
HOMO	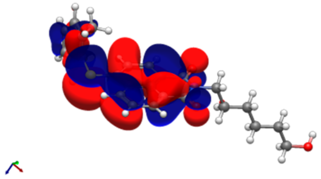	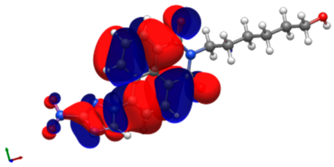	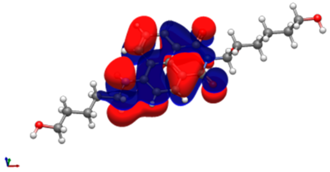
HOMO-1	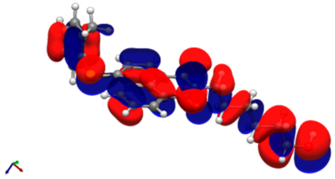	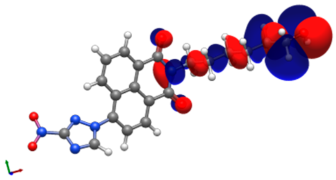	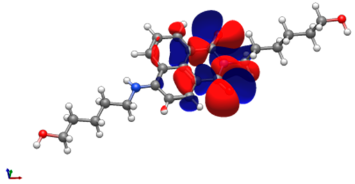
HOMO-2	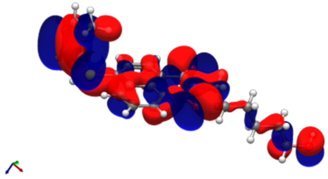	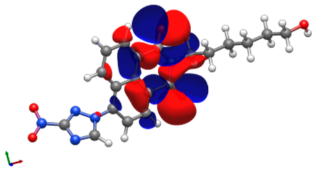	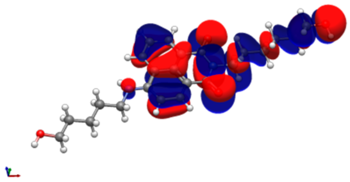
